# Evaluating deconvolution methods using real bulk RNA-expression data for robust prognostic insights across cancer types

**DOI:** 10.1186/s13059-026-03942-1

**Published:** 2026-01-21

**Authors:** Minghan Li, Yuqing Su, Yizhou Tang, Yuehfan Lee, Weidong Tian

**Affiliations:** 1 State Key Laboratory of Genetic Engineering, Department of Computational Biology, School of Life Sciences, Fudan University, Shanghai, China; 2 Children’s Hospital of Fudan University, Shanghai, China; 3 Children’s Hospital of Shandong University, Jinan, China

## Abstract

**Background:**

Deconvolution of bulk RNA-expression data unlocks the cellular complexity of cancer, yet traditional pseudobulk benchmarks may not always be reliable in real-world settings where absolute cell proportions are unknown.

**Results:**

Here, we introduce a novel real-data framework, leveraging 18 real bulk RNA-expression cohorts (5,891 samples) across nine cancer types to evaluate five deconvolution methods based on differentially proportioned (DP) and prognosis-related (PR) cell types. Across three innovative benchmark scenarios—consistency with scRNA-seq, reproducibility across cohorts, and reproducibility of prognostic relevance—ReCIDE and BayesPrism stand out as two robust deconvolution methods. Application of a pan-cancer analysis based on the deconvolution of TCGA cohorts identifies matrix cancer-associated fibroblasts (mCAF) as a prognostic marker with consistent effects across multiple cancers. Building on this finding, we find a prognostic indicator combining classical monocytes and mCAF cell proportions to be significant in five TCGA cohorts, which we further validate in five independent GEO cohorts.

**Conclusions:**

This study broadens deconvolution benchmarking, offering actionable tools for precision oncology and guiding method selection for translational research.

**Supplementary Information:**

The online version contains supplementary material available at 10.1186/s13059-026-03942-1.

## Introduction

The cellular heterogeneity of cancer critically impacts patient outcomes and treatment strategies, yet unraveling this complexity in clinical samples remains a difficult task [1–4]. Single-cell RNA sequencing (scRNA-seq) has begun to pierce this veil, revealing gene expression at unprecedent resolution [5–7], but its cost restricts large-scale studies linking cell types to patient prognosis [8]. Conversly, bulk RNA-seq and microarray data from repositories like TCGA and GEO [9–12], rich with clinical information, offer a scalable alternative. Deconvolution methods bridge these domains, estimating cell type proportions from bulk data using scRNA-seq references to probe disease associations [13, 14].

Yet, a notable challenge persists. Despite advances in methods like CIBERSORT [15], DWLS [16], MuSiC [17], Bisque [18], BayesPrism [19], and our recently developed ReCIDE [20], current benchmarking studies for deconvolution methods invariably lean on pseudobulk data or flow cytometry [21–24], assuming known absolute cell type proportions. In real bulk RNA-expression deconvolution, such precision is a mirage, confounded by biological and technical noise, underminging deconvolution’s clinical utility [25, 26]. To address this, we propose a novel benchmark strategy: evaluating methods based on relative changes in differentially proportioned (DP) cell types—those shifting significantly between conditions—a metric robust across datasets and aligned with clinical research priorities.

Here, we assessed five deconvolution methods across 18 real bulk RNA-expression cohorts from nine cancer types, totalling 5,891 samples using three scenarios: (1) DP cell type alignment with scRNA-seq, (2) reproducibility of DP cell types between cohorts, and (3) reproducibility of prognositic-related (PR) cell types between cohorts. Our analysis suggested that ReCIDE and BayesPrism tend to exhibit robust performance across these benchmarks. Additionally, pan-cancer analyses identified matrix cancer-associated fibroblasts (mCAF) as a prognostic marker with significantly similar effects across multiple cancers. Building on mCAF, we further identified a combination of cell types—classical monocytes and mCAF (classical_mono% + mCAF%)—as a significant prognostic indicator in five TCGA cohorts, which was further validated across five independent GEO cohorts. This real-data approach not only refines method selection but also advances precision oncology by linking cellular insights to patient outcomes.

## Results

### Benchmark design

**Fig. 1:**
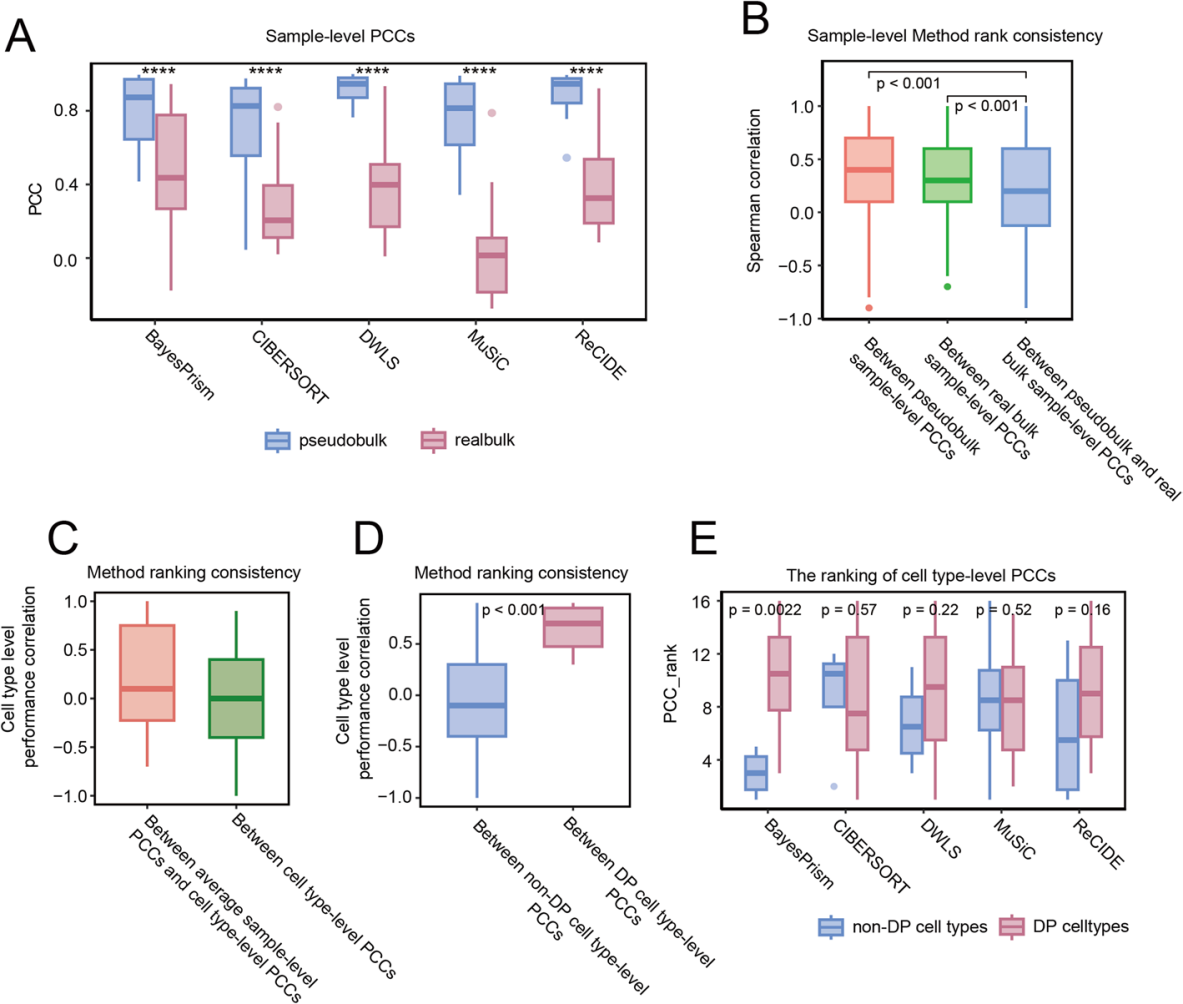
Rationale for evaluating differentially proportioned (DP) cell types. **A** Sample-level PCCs across pseudobulk and real bulk cohorts. **B** Consistency of method rankings within pseudobulk and real bulk cohorts, and between these two data types. **C** Consistency of method rankings between average sample-level PCCs and cell type-level PCCs, and across different cell types. **D** Consistency of method rankings between DP and non-DP cell types in real bulk cohorts. **E** Rankings of cell type-level PCCs for DP and non-DP cell types in real bulk cohorts. In panels (**B**-**D**), ranking consistency was quantified using Spearman correlation

Traditional benchmarks of deconvolution methods typically rely on pseudobulk expression data. However, accumulating evidence indicates that pseudobulk and real bulk RNA-seq represent distinct modalities, and strong performance on pseudobulk does not necessarily translate to reliable performance on real bulk data [25, 26]. We confirmed this discrepancy using a paired breast cancer dataset (GSE176078 [27]), in which each sample was profiled by both scRNA-seq and bulk RNA-seq. Pseudobulk profiles were generated with SimBu [28], and deconvolution accuracy was assessed using scRNA-seq-derived ground truth proportions. As expected, pseudobulk consistently produced higher sample-level Pearson correlation coefficients (PCC) than real bulk (Fig. 1A, Methods). Moreover, method performance rankings were less consistent between pseudobulk and real bulk than within either data type alone (*p* < 0.001, Fig. 1B, Table S1). This finding suggests that rankings derived from pseudobulk may not be directly transferable to real bulk settings and underscores the necessity of evaluating methods directly on real bulk data. We further observed that commonly used metrics such as averaged sample-level PCC obscure substantial heterogeneity in cell-type-specific performance (Fig. 1C).

To address these limitations, we focused our evaluation on differentially proportioned (DP) cell types, defined by condition-level changes rather than sample-level variation. Because DP cell types reflect disease-relevant biological contrasts, they provide a more robust basis for benchmarking. In the GSE176078 dataset, which contains two conditions (ER + breast cancer and TNBC), four DP cell types (Monocyte_Macrophage, Cycling_Myeloid, DCs, and Endothelial_CXCL12) were identified from scRNA-seq data using a Wilcoxon test (FDR < 0.2). These DP cell types exhibited significantly higher method ranking consistency (*p* < 0.001) and generally greater estimation accuracy than non-DP cell types in real bulk data (Fig. 1D, E, Fig. S1). Thus, focusing on DP cell types reduces noise from irrelevant variation while highlighting disease-related signals.

Importantly, DP cell types can be identified using condition-level differences without requiring matched scRNA-seq and bulk data, and they are expected to remain consistent across cohorts with the same disease contrast. This enables reproducibility to be assessed directly from bulk datasets. Building on this rationale, we designed three benchmark scenarios. The first evaluates the consistency of DP cell types between deconvolution results and scRNA-seq data. The second assesses the reproducibility of DP cell types across independent bulk cohorts. The third extends the concept by defining prognosis-related (PR) cell types, whose proportions are significantly associated with survival, and evaluates their reproducibility across cohorts where survival data are available. Together, these scenarios address both biological relevance and clinical utility, aligning the benchmarks with translational research priorities.

**Fig. 2:**
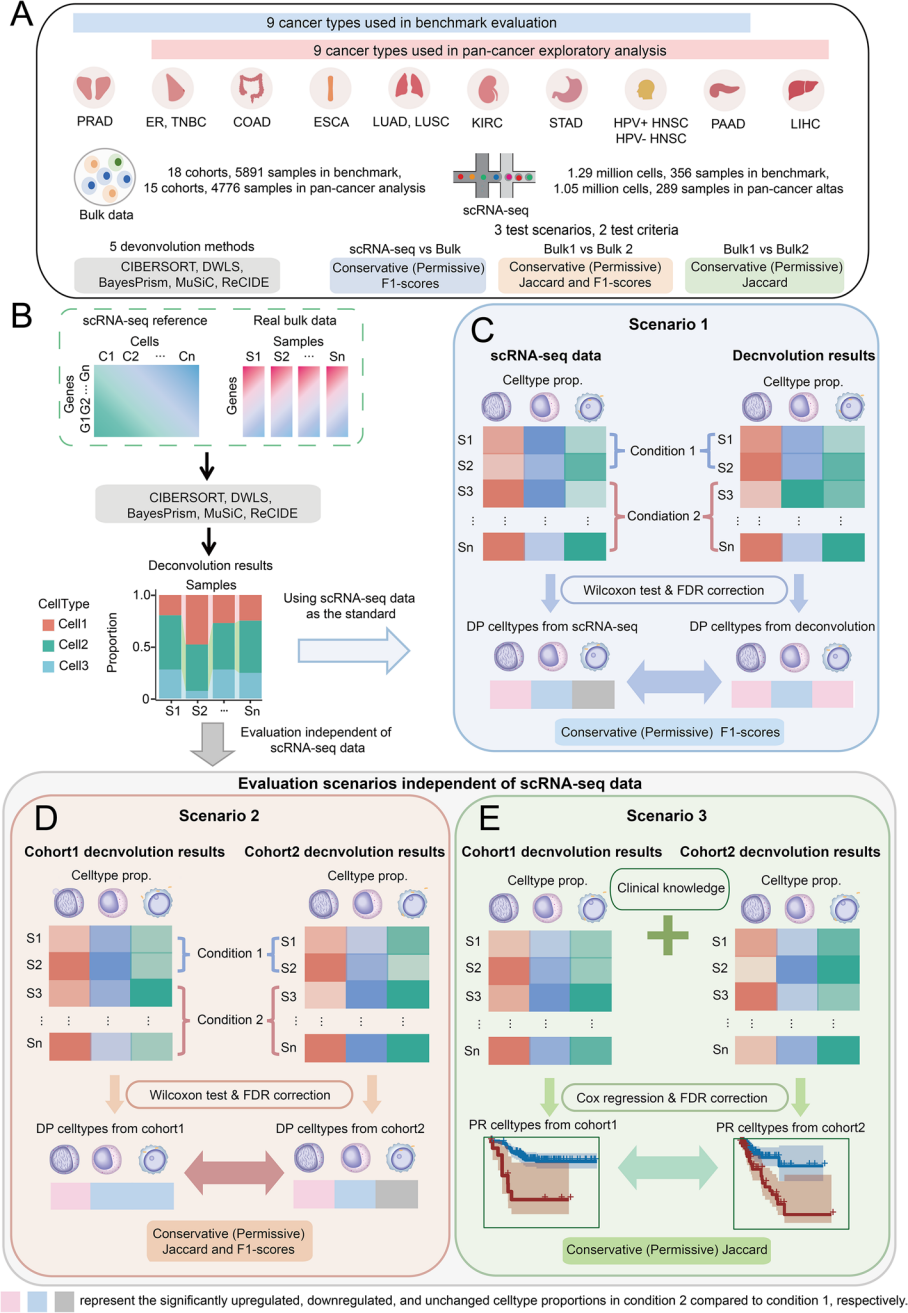
Schematic representation of the benchmarking study and the experimental design of our research. **A** Summary of datasets, deconvolution methods, and evaluation metrics involved in this study. **B**-**E** Illustration of the three evaluation scenarios. **C** Scenario 1 assesses consistency of differentially proportioned (DP) cell types between deconvolution results and scRNA-seq data. **D** Scenario 2 evaluates reproducibility of DP cell types across TCGA and GEO cohorts. **E** Scenario 3 examines reproducibility of prognosis-related (PR) cell types across TCGA and GEO cohorts

For benchmarking, we selected five widely used deconvolution methods that had previously ranked among the top performers (CIBERSORT [15], DWLS [16], MuSiC [17], Bisque [18], and BayesPrism [19]) (Cobos et al. [22], 2020; Chu et al. [19], 2022) and incorporated our newly developed method, ReCIDE [20] (Fig. 2A, B). During preliminary analyses, we found that Bisque exhibited reference-dependent bias: its results varied substantially with shifts in cell type proportions in the scRNA-seq reference, a behavior not observed in other methods (Fig. S2). Because this bias could confound assessments of DP consistency across cohorts, Bisque was excluded from all subsequent analyses.

We initially assembled datasets from 10 cancer types representing 13 cancer entities (including subtypes), with each cancer type comprising one scRNA-seq cohort and two bulk cohorts (one from GEO and one from TCGA), all corresponding to the same disease contrast conditions. These contrast conditions include tumor vs. adjacent normal tissue (for seven cancers) and cancer subtypes (for three cancers). For DP and PR-based evaluations, eight cancer types and five cancer entities were selected, based on sample size or available survival information (Table S2, Methods). Detailed information on data acquisition and DP/PR cell type identification is provided in the "Methods" and Data Availability section.

### Consistency of DP cell types between deconvolved bulk and scRNA-seq data

**Fig. 3:**
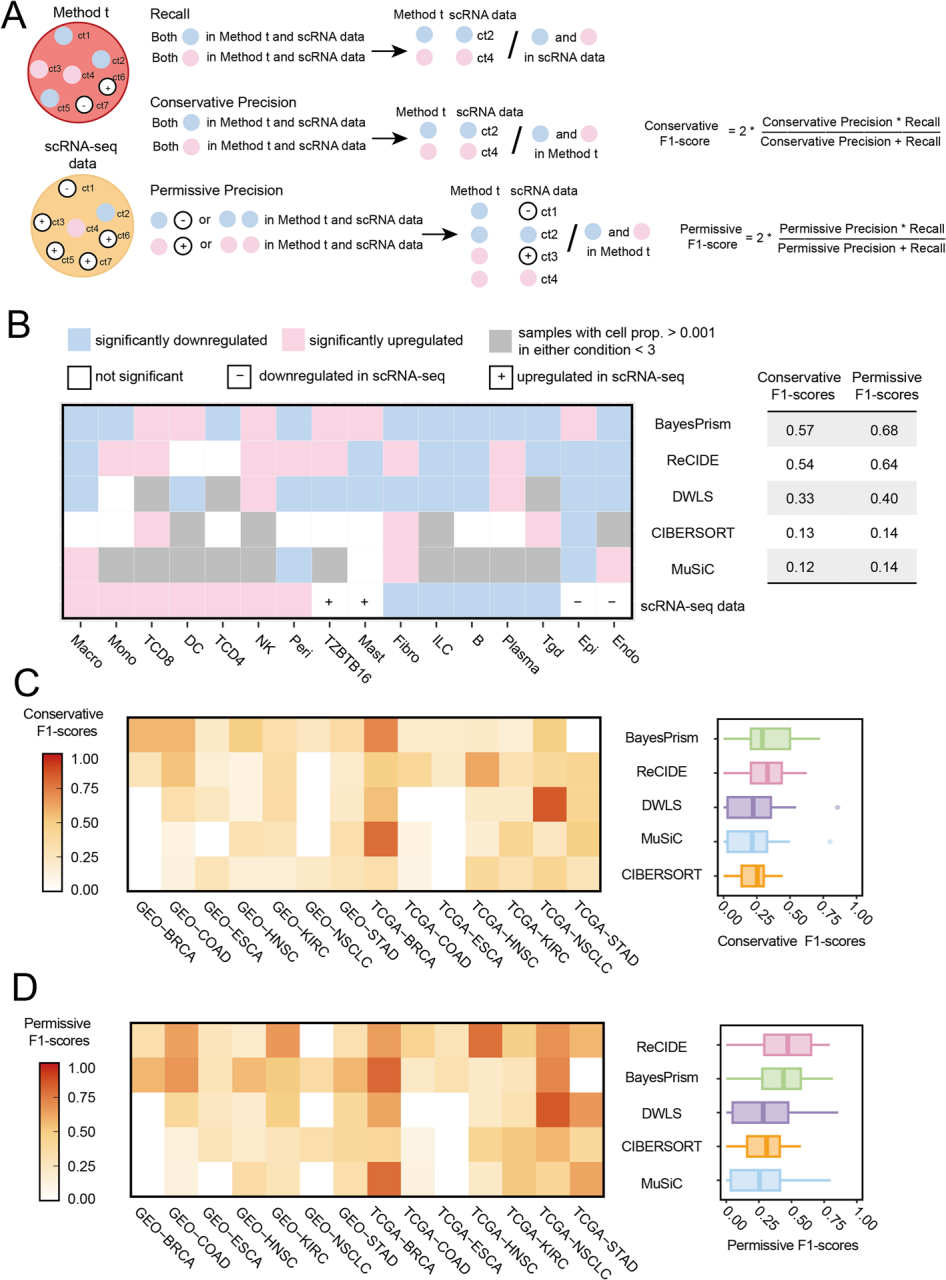
The consistency between the DP cell types identified by the deconvolution methods and the scRNA-seq reference. **A** Detailed description of the calculation for conservative and permissive F1-score. **B** Detailed representation of conservative and permissive F1-score within the GEO-COAD dataset. **C**, **D** Results of the consistency between deconvolution results and scRNA-seq standard across eight cancer types for five methods. PRAD was excluded because no DP cell types were detected in its scRNA-seq data. **C** Conservative F1-scores. **D** Permissive F1-scores

We assessed how well deconvolution methods identify differentially proportioned (DP) cell types in real bulk expression data by comparing their results with those defined by scRNA-seq, using five methods. The F1-score served as the primary evaluation metric. We applied both conservative and permissive criteria to account for discrepancies in sample sizes between scRNA-seq and bulk cohorts (Fig. 3A). Under conservative criteria, precision represents the fraction of deconvolution-identified DP cell types that are confirmed in scRNA-seq, whereas under permissive criteria, precision additionally includes cell types whose proportional changes exhibit trends consistent with the deconvolution results, even if they are not statistically DP in scRNA-seq. Recall measures the proportion of scRNA-seq DP cell types recovered by deconvolution. The calculations for recall, precision, and F1-score are detailed in the "Methods" section.

We began by analyzing colon adenocarcinoma (COAD), which includes a large scRNA-seq cohort of 35 normal and 53 tumor samples with 19 cell types. For the evaluation, we excluded three cell types with extremely low proportions in either condition (Methods). From the scRNA-seq dataset, we identified 12 DP cell types, including seven enriched and five reduced in tumors (Fig. 3B). While deconvolution methods showed variations in absolute proportions (e.g., median proportion of B cells in tumor vs. normal tissue: 0.031 vs. 0.10 in scRNA-seq vs. BayesPrism 0.087 vs. 0.171, DWLS 0.135 vs. 0.184, ReCIDE 0.176 vs. 0.184 in GEO-COAD; Fig. S3), the relative trends from BayesPrism, DWLS, and ReCIDE closely matched scRNA-seq findings. These methods consistently detected a significant downregulation of B cells in tumors, demonstrating their ability to capture proportional shifts even when absolute values differed.

Next, we assessed F1-scores to evaluate consistency between deconvolution methods and scRNA-seq-defined DP cell types. Under conservative criteria, BayesPrism achieved the highest F1-score of 0.57 (8/16 DP cell types validated; recall 0.67, precision 0.5), followed by ReCIDE at 0.54 (7/14 validated; recall 0.58, precision 0.5). MuSiC lagged behind with a low F1-score (0.12, recall = 0.083, precision = 0.2). Under permissive criteria, all methods showed improved F1-scores. For example, BayesPrism's F1-score increased to 0.68 (precision = 0.69), ReCIDE's to 0.64 (precision = 0.71), and MuSiC's to 0.14 (precision = 0.4).

We also observed that MuSiC and CIBERSORT frequently assigned zero proportions to some cell types, such as NK cells in the GEO-COAD cohort, where MuSiC and CIBERSORT assigned zero proportions to 99.0% and 81.9% of samples, respectively (Fig. S4). To avoid bias from overestimating consistency due to unsuccessful deconvolution, we excluded samples where the estimated proportion of a cell type was below 0.1% when evaluating the significance of proportion changes (Methods). This exclusion likely contributed to the lower consistency observed between MuSiC, CIBERSORT, and scRNA-seq trends.

Expanding our analysis to eight cancer types, BayesPrism and ReCIDE demonstrated superior consistency with scRNA-seq-derived DP cell types (Fig. 3C, D). These two methods showed minimal differences between the two metrics (0.35 vs. 0.33 for conservative F1-score, 0.44 vs. 0.46 for permissive F1-score), both outperforming DWLS, the third-ranked method, by over 15% in both metrics. In contrast, MuSiC and CIBERSORT had lower F1-scores, with conservative F1-scores of 0.24 and 0.22, and permissive F1-scores of 0.28 and 0.29, respectively, reflecting their limitations in handling heterogeneous data within this framework. The average F1-scores of most methods were higher under permissive conditions, as deconvolution-defined DP cell types were easier to validate under these criteria, leading to higher precision and F1-scores (Fig. S5).

To evaluate the impact of minor DP cell types (defined as those with < 1% abundance in either condition in scRNA-seq), we repeated the analysis excluding these cell types. The ranking performance remained largely consistent with the full analysis (Fig. S6), demonstrating the robustness and stability of the DP-based benchmark evaluation.

In summary, BayesPrism and ReCIDE are the top performers in this benchmark scenario. Their consistent alignment with scRNA-seq data far surpasses that of other methods, highlighting their higher clinical utility.

### Reproducibility of DP cell types across deconvolved bulk cohorts

Assessing the reproducibility of differentially proportioned (DP) cell types across bulk cohorts with matching disease conditions is crucial for real-world applicability. This was demonstrated by Fonseca et al.’s validation [29] of ciliated endometrial-type epithelial cells enrichment in multiple ovarian cancer cohorts using MuSiC. In this benchmark analysis, we evaluated the reproducibility of DP cell types across paired bulk cohorts using five deconvolution methods.

**Fig. 4:**
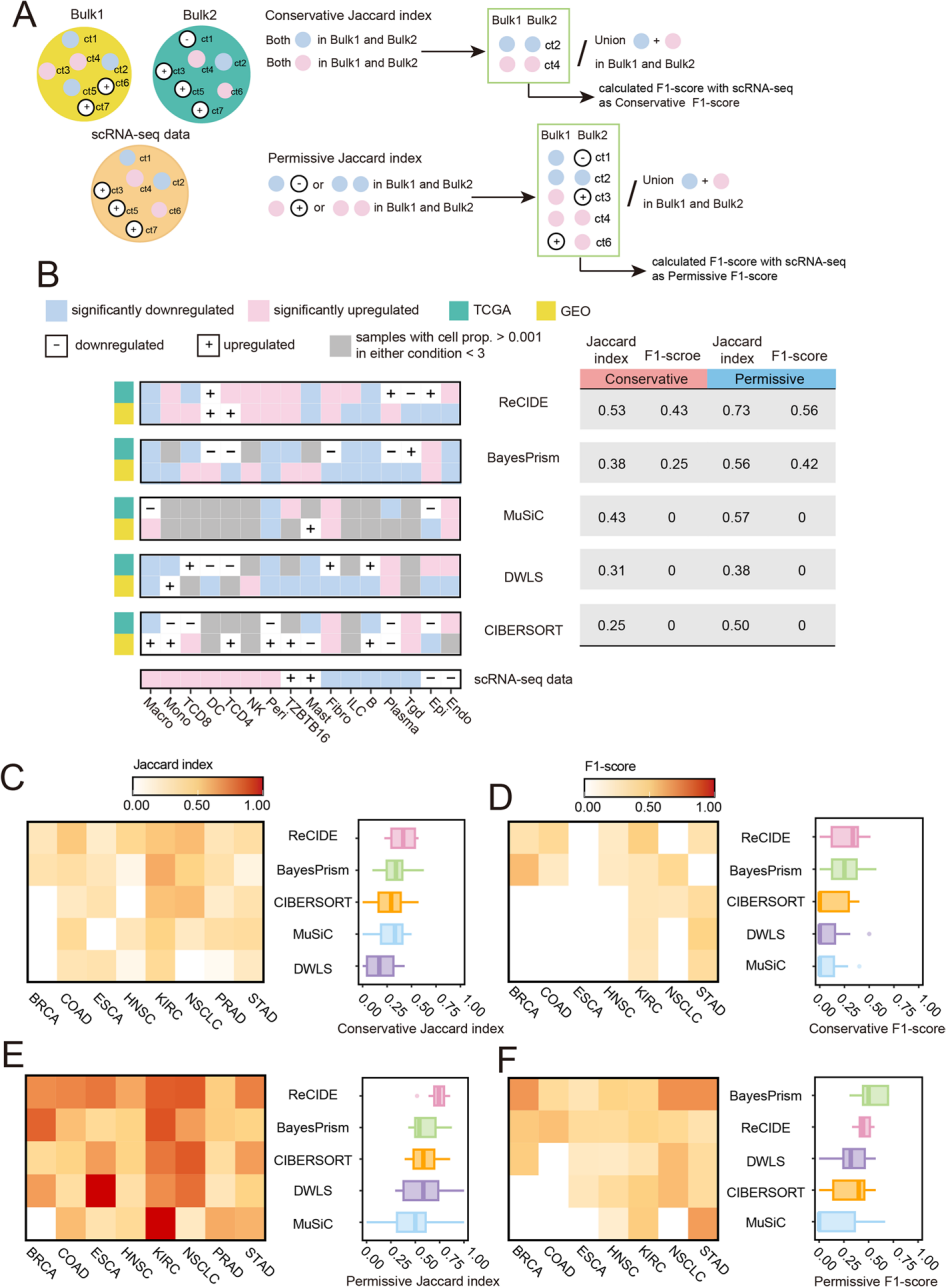
Reproducibility of DP cell type identification across cohorts. **A** Detailed description of the calculation for conservative and permissive Jaccard indices. **B** Detailed representation of conservative and permissive Jaccard indices for five methods in COAD (TCGA and GEO cohorts). **C**-**F** Results of the reproducibility and consistency across eight cancer types for five methods. **C** Conservative Jaccard index. **D** Conservative F1-scores for shared DP cell types vs. scRNA-seq. **E** Permissive Jaccard index. **F** Permissive F1-scores for shared DP cell types vs. scRNA-seq

Reproducibility was measured using the Jaccard index (Jaccard), which calculates the overlap of DP cell types between paired cohorts. Accuracy was assessed using the F1-score, which compares shared DP cell types to those defined by scRNA-seq (Fig. 4A). We applied both conservative and permissive criteria: conservative criteria required statistical significance in both cohorts for a shared DP cell type, while permissive criteria accepted a significant association in one cohort with a consistent trend in the other. For F1-scores, precision is the fraction of shared DP cell types matching scRNA-seq among all discovered shared DP cell types, while recall measures the proportion of scRNA-seq DP cell types recovered among shared ones, balancing both metrics (Methods).

We first assessed reproducibility in colon adenocarcinoma (COAD), where 12 DP cell types were identified from the scRNA-seq dataset. Under conservative criteria, ReCIDE detected 15 DP cell types across TCGA and GEO cohorts, with 8 shared (Jaccard = 0.53) (Fig. 4B). Of these eight, five showed consistent trends with scRNA-seq in which four exhibited significant changes in scRNA-seq, resulting in an F1-score of 0.43 (recall = 0.33, precision = 0.63). BayesPrism identified 16 DP cell types, with 6 shared (Jaccard = 0.38), in which three showed consistent trends with scRNA-seq and one is a DP in scRNA-seq, yielding an F1-score of 0.25 (recall = 0.17, precision = 0.5). CIBERSORT identified eight DP cell types, but only two overlapped (Jaccard = 0.25), with no consistent trends (F1-score = 0). Under permissive criteria, ReCIDE identified three additional shared DP cell types (Jaccard = 0.73), increasing the number of aligned cell types to seven, yielding an F1-score of 0.56 (recall = 0.5, precision = 0.64). BayesPrism added three more shared cell types (Jaccard = 0.56), with five aligned trends, yielding an F1-score of 0.42 (recall = 0.33, precision = 0.56). CIBERSORT’s Jaccard index increased to 0.5, but its F1-score remained at 0.

Across eight cancer types, ReCIDE ranked first with Jaccard index of 0.41 (conservative) and 0.73 (permissive), demonstrating the strongest cross-cohort reproducibility (Fig. 4C, E), followed by BayesPrism (0.34 and 0.61, respectively). As for F1-score, ReCIDE is slightly bettet than BayesPrism under conservation criteria (0.27 vs 0.26, Fig. 4D), while BayesPrism outperformed ReCIDE in permissive criteria (0.54 vs 0.46, Fig. 4F). MuSiC and DWLS underperformed in various metrics, with CIBERSORT ranking fourth in F1-score under permissive criteria and third in the other metrics. Similar to scenario one, the F1-scores of most methods improved under the permissive criteria primarily due to the increased number of shared cell type, which led to higher recall and precision (Fig. S7).

In summary, ReCIDE and BayesPrism exhibited superior performance in the reproducibility benchmark, with ReCIDE excelling in consistency across cohorts and BayesPrism showing stronger alignment with scRNA-seq data.

### Reproducibility of prognosis-related cell types across deconvolved bulk cohorts

Identifying prognosis-related (PR) cell types that correlate with cancer patient survival is crucial for patient stratification and the development of targeted therapies. We selected five cancer entities—COAD, HPV-HNSC, LUAD, LUSC, and PAAD—all with paired cohorts that include survival data (one from TCGA and one from GEO). LUAD and LUSC are subtypes of NSCLC, and HPV + HNSC is a subtype of HNSC. These five cancer entities were used to evaluate the reproducibility of five deconvolution methods in detecting PR cell types across paired cohorts.

**Fig. 5:**
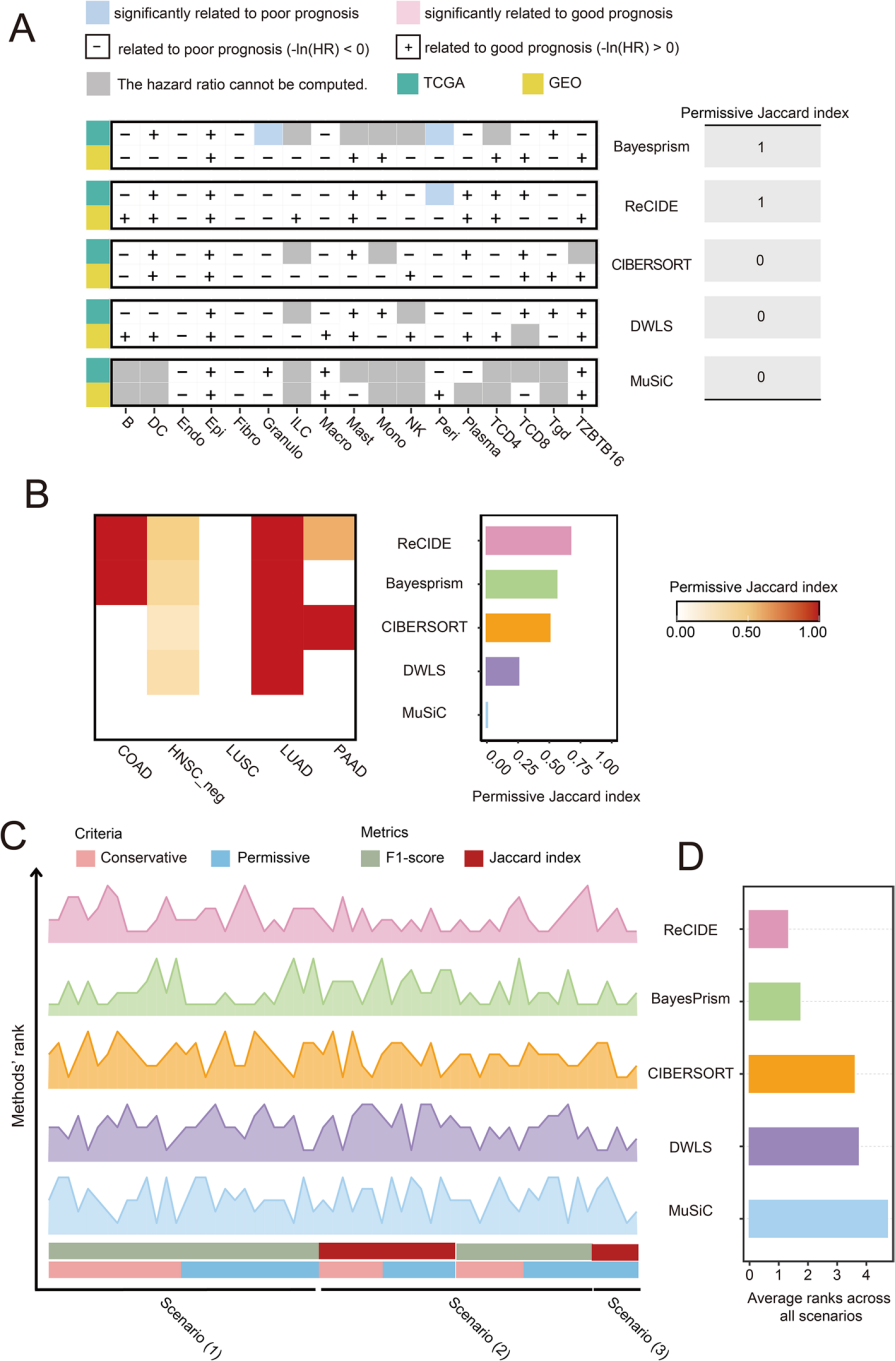
Reproducibility of PR cell type identification across cohorts and overall method performance. **A** Detailed representation of conservative and permissive Jaccard for five methods in COAD (TCGA and GEO cohorts). **B** Permissive Jaccard index across five cancer entities for five methods. **C** Detailed rankings of the five deconvolution methods across all scenarios and metrics. **D** Average rankings of the five deconvolution methods across all scenarios

Reproducibility was assessed using both conservative and permissive criteria. Conservative criteria required statistical significance for a shared PR cell type in both cohorts, while permissive criteria accepted significance in one cohort if the trend was consistent in the other. Multivariate Cox regression was performed to identify PR cell types (Methods). In the COAD dataset (Fig. 5A), BayesPrism identified two PR cell types in the TCGA cohort and ReCIDE identified one, but neither identified any in the GEO cohort. However, under permissive criteria, all PR cell types identified by both methods were validated in the GEO cohort.

Given the limited number of PR cell types (usually one or a few per cohort), calculating the Jaccard index for each cancer entity individually resulted in unstable reproducibility estmiates. Therefore, we combined PR cell types across all five cancer entities to compute a more stable Jaccard index (Fig. 5B). Under conservative criteria, no method identified a shared PR cell type that was significant in both cohorts across the five cancer types. Under permissive criteria, most methods detected shared PR cell types in at least two cancer types. ReCIDE achieved the highest Jaccard index of 0.67, followed by BayesPrism at 0.56. CIBERSORT and DWLS had Jaccard indices of 0.5 and 0.25, respectively. MuSiC performed poorly, failing to identify shared PR cell types in any cancer type. When we relaxed the false discovery rate (FDR) threshold for identifying PR cell types (from 0.05 to 0.10 and 0.15), ReCIDE and BayesPrism maintained their top positions, with other methods showing little change in ranking (Fig. S8).

In summary, ReCIDE and BayesPrism outperformed other methods in consistently identifying PR cell types across cohorts, underscoring their value for deconvolution-based prognostic analysis.

### Comprehensive performance across three evaluation scenarios

We evaluated five deconvolution methods across three scenarios: (1) consistency of differentially proportioned (DP) cell types with scRNA-seq (Scenario 1), (2) reproducibility of DP cell types across bulk cohorts (Scenario 2), and (3) reproducibility of prognosis-related (PR) cell types (Scenario 3). Each method was assessed using the F1-score or Jaccard index under both conservative and permissive criteria, with detailed results provided in previous subsections. To summarize overall performance, we averaged the ranks of each method across these metrics (Fig. 5C, D).

ReCIDE emerged as the top performer, with an average rank of 1.29, demonstrating its robust ability to detect and reproduce both DP and PR cell types. BayesPrism closely followed, with an average rank of 1.71, often ranking near the top in various scenarios. In contrast, the average ranks of CIBERSORT, DWLS, and MuSiC were 3.57, 3.71, and 4.71, respectively, across these benchmarks.

### Pan-cancer prognostic analysis based on deconvolution results

**Fig. 6:**
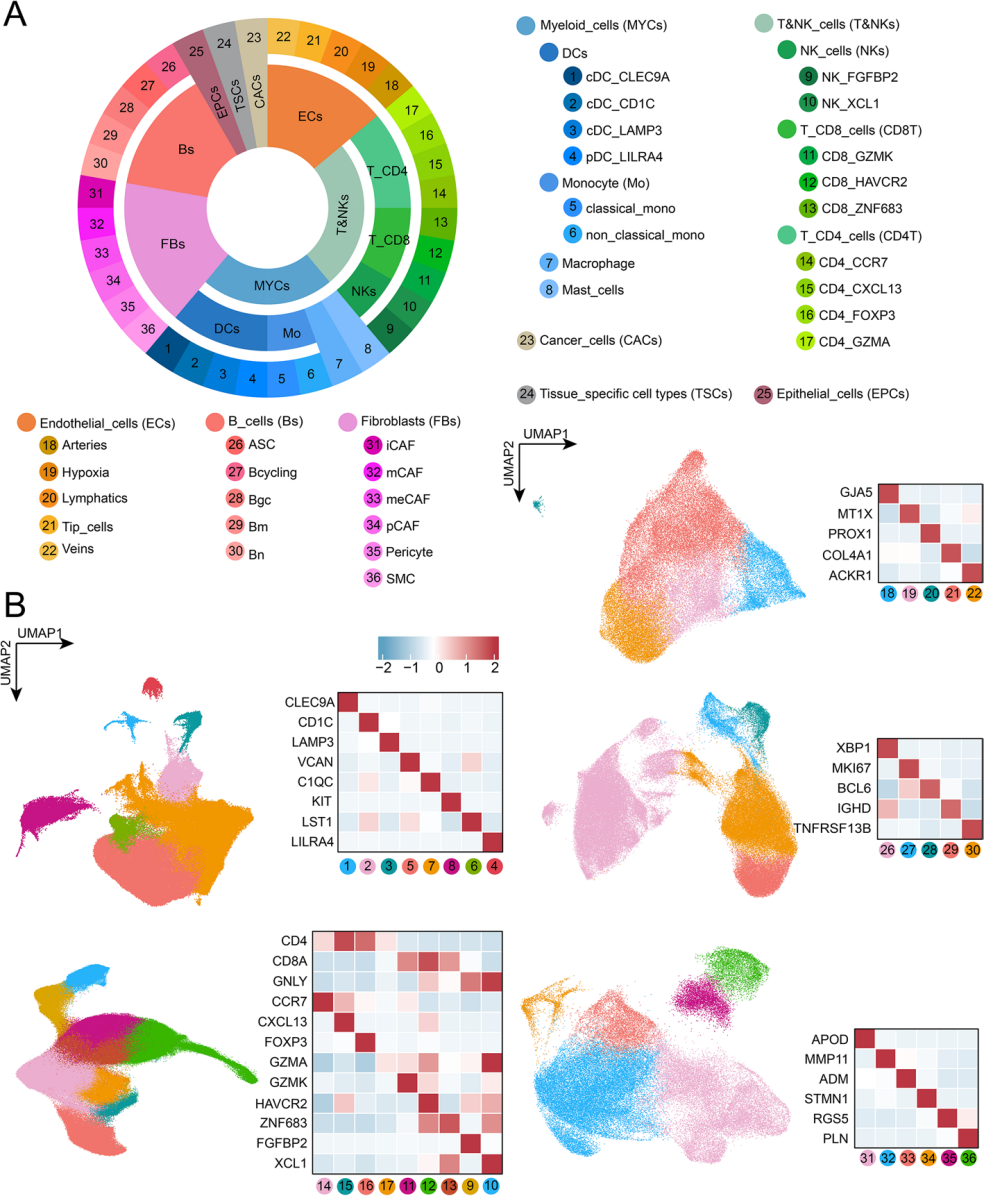
Pan-cancer cell atlas composition. **A** UMAP visualization of the atlas, featuring eight major cell types and 36 subtypes (nine T & NK_cells, eight myeloid_cells, six fibroblasts, five endothelial_cells, five B_cells, three others). **B** The composition of cell subtypes within each major cell type, with marker genes listed for annotation

We constructed a pan-cancer single-cell atlas by aggregating scRNA-seq data from 10 cancer types (Methods). Cells were classified into eight major types based on their original annotations: epithelial cells, cancer cells, tissue-specific cells, and five immune and stromal cell types (T & NK cells, myeloid cells, fibroblasts, endothelial cells, and B cells). The five major immune and stromal types were further clustered and reannotated using published marker genes to harmonize subtypes across datasets, yielding 33 subtypes (Fig. 6). These subtypes, together with the three unsplit major cell types, were used for pan-cancer deconvolution analyses.

**Fig. 7:**
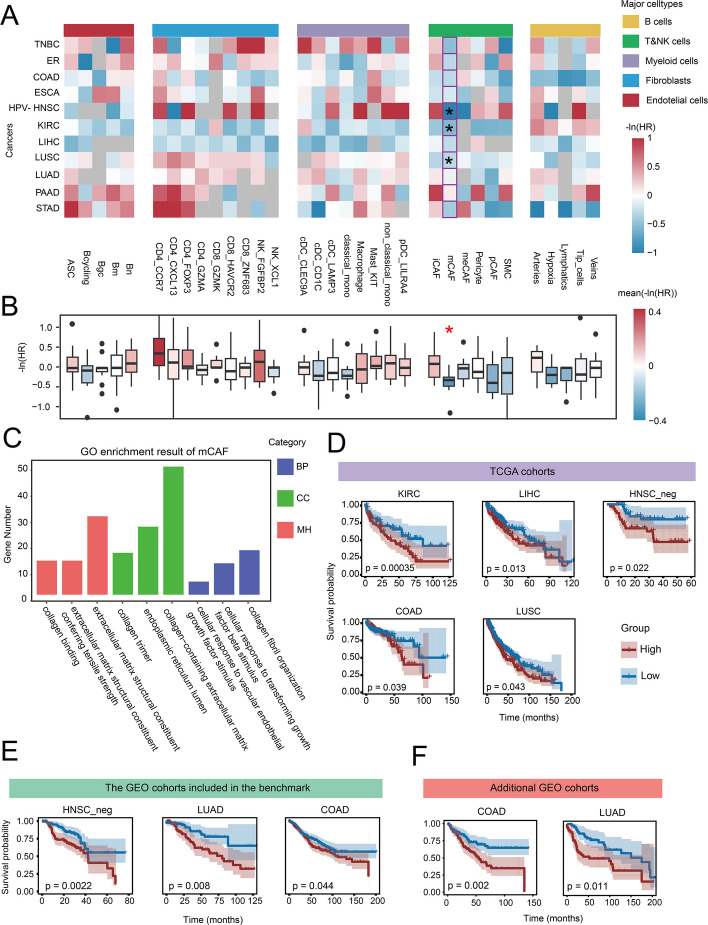
Pan-cancer prognostic analysis results. **A** Heatmap of prognostic correlations between 33 cell subtypes and survival across 11 cancer entities, missing values indicate that, for the corresponding cell type, the number of deconvolution results with a cell proportion greater than 0.1% was insufficient to support survival analysis (with fewer than 15 death events). Results with -ln(HR) values greater than 1 or less than −1 are displayed as 1 and −1, respectively. **B** Boxplot of -ln(HR) values across 11 cancer entities, with significant pan-cancer prognostic cell types (one-sided Wilcoxon Signed-Rank Test, FDR-corrected) highlighted. **C** GO enrichment results of mCAFs marker genes. **D**-**F** Kaplan–Meier survival curves in TCGA and GEO cohorts for prognostic indicators, p values were derived from multivariate Cox regression. **D** TCGA cohorts. **E** GEO cohorts used in benchmark. **F** Additional GEO cohorts (GSE17536 and GSE30219)

We selected 11 cancer entities with available TCGA bulk cohorts and survival data (PRAD and HPV + HNSC were excluded because the number of death events was less than 15). We applied ReCIDE and BayesPrism to deconvolve these cohorts using their respective scRNA-seq references with unified cell type label. Multivariate Cox regression was performed to assess the relationship between cell subtype proportions and patient survival outcomes (Methods). Hazard ratios (HR) were calculated, where -ln(HR) > 0 indicates a favorable prognosis and -ln(HR) < 0 denotes a poor prognosis (Fig. 7A). A one-sided Wilcoxon Signed-Rank Test with FDR correction identified cell subtypes with significantly consistent prognostic effects across the 11 cancer entities (Fig. 7B).

ReCIDE identified matrix cancer-associated fibroblasts (mCAF) as significantly correlated with pan-cancer prognosis (*p* = 0.032), showing a significant negative correlation with survival in ER + BC, HPV- HNSC, and KIRC, and a similar trend in seven other cancer entities (Fig. 7A, Fig. S9). BayesPrism did not identify any cell subtype with significant consistency in prognostic effects across pan-cancer cohorts (Fig. S10).

To understand why mCAF was significantly associated with prognosis, we identified genes specifically expressed in mCAF compared to other five CAF subtypes and performed GO enrichment analysis. mCAF was enriched in pathways related to "cellular response to TGF-beta stimulus" and "cellular response to VEGF stimulus" (Fig. 7C), suggesting that mCAF contributes to angiogenesis, supporting tumor growth and metastasis [30].

Building on the mCAF finding, we explored whether combining mCAF with other cell types could serve as a pan-cancer prognostic indicator. Combining mCAF with classical monocyte proportions significantly correlated with survival in five of the 11 TCGA entities (Fig. 7D, Fig. S11A). We validated this indicator in five independent GEO cohorts from five cancer entities (including LUAD and LUSC as subtypes of NSCLC), finding it significant in three entities (Fig. 7E, Fig. S11B). Further validation in two additional GEO cohort—one COAD cohort (GSE17536 [31]) and one LUAD cohort (GSE30219 [32])—also showed significant negative correlations with prognosis (p_LUAD_ = 0.002, p_LUAD_ = 0.011) (Fig. 7F). These results suggested the reliability of the prognostic indicator for patient stratification across multiple cancer types, underscoring its clinical utility in pan-cancer prognostic studies.

## Discussion

Deconvolution methods are essential for analyzing the cellular composition of tumor microenvironments, with widespread applications in cancer research [33, 34]. While earlier studies used pseudobulk data for performance evaluation, recent research highlights that strong pseudobulk performance does not necessarily guarantee reliable results with real bulk data [25, 26]. To address this, we introduced a novel evaluation framework that emphasizes the consistency and reproducibility of differentially proportioned (DP) cell types across disease conditions in real bulk cohorts.

Our central hypothesis posits that DP cell types, being biologically relevant and mechanism-driven, should replicate across cohorts. While complete replication may be unlikely due to factors like patient heterogeneity and batch effects, substantial replication is expected as cohorts share similar disease conditions and underlying biological mechanisms. Although cohort differences may influence DP cell proportions, this variability may affect all methods similarly, ensuring the validity of evaluating method consistency. Our benchmark results show that core biological signals can be reliably captured, even amidst cohort-specific variability, validating the robustness of our evaluation framework.

In our analysis, we emphasized the consistency and reproducibility of DP cell types without considering the magnitudes of proportional changes. This decision stemmed from the concern that such magnitudes are often affected by batch effects and variations in cell composition across bulk cohorts. To assess this, we conducted a median absolute error (MAE) analysis comparing log2 fold changes (log2FC) of DP cell types. MAE analysis revealed that in Scenario one, BayesPrism, ReCIDE, and CIBERSORT ranked highest, while in Scenario two, ReCIDE, CIBERSORT, and BayesPrism led the rankings (Fig. S12). These variations reflect the differing contexts of the scenarios: Scenario 1 compares detailed scRNA-seq data to bulk RNA-seq signals, while Scenario 2 compares two bulk RNA-seq datasets, subject to batch effects and cell composition variations. In contrast, our approach, focusing on the presence or absence of DP cell types, consistently ranked methods across both scenarios, suggesting that our qualitative approach is currently more reliable and robust than focusing solely on quantitative accuracy.

While our benchmarks primarily focused on cancer datasets, we extended our analysis to non-cancer diseases like COVID-19 and systemic lupus erythematosus (SLE), where global shifts in cellular composition are less pronounced. We applied the same deconvolution framework to four non-cancer datasets (GSE161731 [35], GSE202805 [36] for COVID-19; GSE121239 [37], GSE81622 [38] for SLE), assessing the consistency and reproducibility of DP cell types across cohorts and comparing them to scRNA-seq data **(**Fig. S13). Our findings, including F1-score and Jaccard index, mirrored those from cancer data: ReCIDE and BayesPrism showed better performance than other methods, highlighting the utility of our DP-based framework in a broader disease context. However, further benchmarks will be needed for a more comprehensive evaluation.

Across all benchmark scenarios, ReCIDE and BayesPrism tended to exhibit distinct advantages, while MuSiC showed limited performance with real-world data, potentially due to its sensitivity to noise. Further analysis of scenario one’s results, separated by microarray and RNA-seq cohorts, revealed some differences between the platforms (Fig. S14). However, both ReCIDE and BayesPrism consistently consistently showed solid performance on either platform, underscoring the robustness of their performance.

In conclusion, our framework provides a real-data-driven evaluation of deconvolution methods that may mitigate some limitations of pseudobulk approaches. This work contributes to the ongoing effort to refine deconvolution tools in translational cancer research and offers a potentially robust method for evaluating performance across diverse biological datasets.

## Conclusion

Previous studies have noted that pseudobulk RNA-seq benchmarks may not fully reflect deconvolution performance in real bulk data, where biological heterogeneity and cohort effects are more pronounced. Using representative examples, our analyses further illustrate that performance metrics and method rankings derived from pseudobulk settings do not always translate to real datasets. To address this gap, we evaluated deconvolution methods using a framework centered on differentially proportioned (DP) cell types, which capture reproducible cellular changes across biological conditions. This benchmark emphasizes consistency between bulk and scRNA-seq data, reproducibility across independent bulk cohorts, and robustness of prognosis-related (PR) cell types in datasets with survival information. Incorporating PR cell types provides a clinically relevant evaluation axis for assessing method robustness, with consistent survival associations supported by existing biological and clinical literature. Across the scenarios examined, several existing methods show stable and reproducible behavior under DP-based evaluation, indicating that biologically meaningful cellular signals can be reliably recovered when assessed using real data–driven criteria. Together, these results highlight the value of benchmarking strategies that prioritize reproducibility, cross-dataset consistency, and clinical relevance, and provide a practical reference for deconvolution method evaluation in translational genomics.

## Methods

### Data collection

#### Benchmark and pan-cancer studies

##### scRNA-seq data

We collected scRNA-seq datasets from 10 cancer types that met the following criteria: (1) each dataset included more than five samples, (2) samples represented two disease contrast conditions (e.g., tumor vs. adjacent normal or distinct cancer subtypes), and (3) cell type annotations were provided by the original authors. The cancer types included were breast cancer (BRCA) [39], colon adenocarcinoma (COAD) [40], esophageal carcinoma (ESCA) [41], head and neck squamous cell carcinoma (HNSC) [42], kidney renal clear cell carcinoma (KIRC) [43], liver hepatocellular carcinoma (LIHC) [44], non-small cell lung cancer (NSCLC) [45], pancreatic adenocarcinoma (PAAD) [46], prostate adenocarcinoma (PRAD) [47], and stomach adenocarcinoma (STAD) [48].

The contrast conditions spanned two categories: (1) tumor vs. adjacent normal tissue for COAD, LIHC, ESCA, KIRC, STAD, and PRAD, and (2) cancer subtype comparisons for BRCA (ER + vs. TNBC), HNSC (HPV + vs. HPV–), and NSCLC (LUAD vs. LUSC).

In our benchmark evaluation, the cell type annotations for the scRNA-seq reference datasets strictly provided by the original authors, without any re-annotation. Detailed information on dataset sources, contrast conditions, sample sizes, and cell type annotations is provided in Tables S3 and S4.

#### Bulk RNA-seq expression data

For each of the 10 cancer types with scRNA-seq data, we obtained two matched bulk cohorts from TCGA and GEO, corresponding to the same primary tumor origin, histopathology, and disease contrast conditions. Cohort sizes ranged from 20 to 885, yielding 20 bulk datasets in total: 12 RNA-seq and 8 microarray [27, 31, 49–57]. Prognostic information was available for 14 cohorts, including all TCGA datasets and 4 GEO cohorts.

In the pan-cancer analysis, we additionally collected one microarray cohort of COAD (GSE17536 [31]) and one of LUAD (GSE30219 [32]) for validation of prognostic indicators.

Detailed information on download sources, disease contrast conditions, sample sizes, and sequencing platforms is provided in Table S2. Survival data were available for all TCGA cohorts and for GEO cohorts of four cancer types (COAD, HNSC, LUCA, and PAAD).

##### Additional studies

To inspect consistency between pseudobulk and real bulk-based estimation, we obtained a paired breast cancer dataset (GSE176078) from GEO. This dataset contains 24 samples with both scRNA-seq and bulk RNA-seq profiles, encompassing three conditions (ER +, TNBC, HER2 +) with 11, 9, and 4 samples, respectively. Only the ER + and TNBC conditions were included in our analysis.

For non-cancer diseases, we analyzed peripheral blood mononuclear cell (PBMC) scRNA-seq data from COVID-19 and systemic lupus erythematosus (SLE). COVID-19 data were obtained from the COVID-19 Cell Atlas (https://covid19cellatlas.org/ [58]) and include 104 samples (81 COVID-19 patients, 23 healthy controls). SLE data were obtained from GSE174188 [59], comprising 251 samples (152 SLE patients, 99 healthy controls). In both diseases, the comparative conditions were healthy versus diseased. Matched bulk cohorts were obtained from GEO: for COVID-19, GSE161731 [35] (77 patients, 16 controls) and GSE202805 [36] (42 patients, 10 controls); for SLE, GSE81622 [38] (15 patients, 25 controls; lupus nephritis samples excluded) and GSE121239 [37], from which we used only the first sequencing run (65 patients, 20 controls).

Comprehensive information on data sources, disease and control conditions, sample sizes for both scRNA-seq and bulk datasets, as well as cell type annotations for the scRNA-seq datasets, is provided in Table S5.

### Overview of deconvolution methods

CIBERSORT was obtained from https://github.com/Moonerss/CIBERSORT and utilized following the instructions provided at https://github.com/Moonerss/CIBERSORT/blob/main/README.md. The inputs included the bulk data and MGM (Marker Gene Matrix). MGM construction began by initially selecting 100 marker genes for each cell type using the cosg() function from the COSG R package [60]. Marker genes with a SecondFC (Second Fold Change) greater than 1.5 were retained [22], and the mean expression values of marker genes for each cell type were utilized to construct the MGM. All other parameters were left at their default settings.

DWLS (R package v0.1.0) was obtained from https://github.com/dtsoucas/DWLS and utilized by following the tutorials provided at https://github.com/dtsoucas/DWLS/blob/master/Manual.docx. Inputs for DWLS included the bulk data, raw counts gene expression matrix of reference scRNA-seq data, and cell type labels. To enhance computational efficiency, the findmarker() function from the Seurat package, invoked by the buildSignatureMatrixUsingSeurat() function in DWLS, was substituted with the cosg() function from the COSG R package. All other parameters were maintained at their default settings.

BayesPrism (R package v2.0) was obtained from https://github.com/Danko-Lab/BayesPrism and utilized by following the tutorial available at https://github.com/Danko-Lab/BayesPrism/blob/main/tutorial_deconvolution.pdf. Inputs for BayesPrism included the bulk data, the raw counts gene expression matrix of reference scRNA-seq data, cell.type.label, and cell.state.label. The annotations of cell.type.label and cell.state.label were processed following Hu et al. [24]. The key parameter in the new.prism() function was configured to account for malignant cells. When malignant cells are not included in the scRNA-seq reference, only cell.type.label is specified. All other parameters remained set to the method's default options.

Bisque (R package v1.0.5) was obtained from https://github.com/cozygene/bisque and utilized by following the guidelines provided in the tutorials available at https://github.com/cozygene/bisque/blob/master/vignettes/bisque.Rmd. Inputs for Bisque included the bulk data, the raw counts gene expression matrix of reference scRNA-seq data, cell type labels, and reference sample labels. All other parameters were configured to the default settings of the algorithm.

MuSiC (R package v1.0.0) was obtained from https://github.com/xuranw/MuSiC and implemented by following the instructions provided in the tutorials at https://xuranw.github.io/MuSiC/articles/MuSiC.html. Inputs to MuSiC included the bulk data, the raw counts gene expression matrix of reference scRNA-seq data, cell type labels, and reference sample labels. All other parameters were left at their default settings.

ReCIDE (R package v1.0.0) was obtained from https://github.com/TianLab-Bioinfo/ReCIDE and implemented by following the instructions provided in the tutorials at https://github.com/TianLab-Bioinfo/ReCIDE/blob/main/README.md. Inputs to ReCIDE included the bulk data, the raw counts gene expression matrix of reference scRNA-seq data, cell type labels, and reference sample labels. All other parameters were left at their default settings.

### Identification of DP and PR cell types

#### Sample and cell type filtering

In some scRNA-seq samples, certain cell types may have extremely low proportions (e.g., < 0.1%) or even be absent, likely due to sequencing artifacts. Including these samples in the identification of DP cell types could lead to false positives. Similarly, some deconvolution methods may assign very low or zero proportions to certain cell types, potentially inflating statistical significance and resulting in false positives. To avoid these biases, we applied the following filtering steps: for scRNA-seq data, we excluded samples where the proportion of a given cell type was below 0.1%. If more than half of the samples in a condition were excluded, the corresponding cell type was removed from DP identification. For deconvolution data, we similarly excluded samples with a cell type proportion below 0.1% when comparing proportional changes. The same filtering process was applied before PR cell type identification.

#### Detection of DP cell types

Of the 10 collected cancer types, LIHC and PAAD were excluded from the DP benchmark: LIHC because its scRNA-seq data were generated by equally mixing immune and non-immune cells, and PAAD because the TCGA cohort included only four adjacent normal samples (Table S2). Consequently, eight cancer types were retained for the DP-based benchmark evaluation.

In the process of identifying DP cells, for tumor and epithelial cells, we applied the cell-type proportion comparison strategy of Lee et al. [61] Specifically, tumor and other epithelial cells were grouped into a single epithelial compartment within each condition, and differences in epithelial proportions were compared across conditions. No merging was performed for other cell types.

To detet DP cell types in either scRNA-seq or bulk cohorts in each of these eight cancer ctypes, we first implemented the filtering step. Then, we conducted a two-sided Wilcoxon test to assess whether the proportions of a given cell type differed significantly between disease contrast conditions, followed by FDR correction. Since scRNA-seq datasets typically have fewer than 20 samples per condition, we set the FDR threshold to 0.1 for identifying DP cell types. For deconvolution-based DP, the FDR threshold was set to 0.05. The details of the identified DP cell types across all cohorts in this study are provided in Table S6.

#### Identification of PR cell types

The 10 collected cancer types corresponded to 13 cancer entities when considering subtypes, five of which—COAD, LUAD, LUSC, HPV⁻ HNSC, and PAAD—had survival information available in matched bulk expression cohorts. These five entities were used to evaluate the reproducibility of PR cell types.

PR cell types were identified using multivariate Cox regression, controlling for confounders where applicable, followed by FDR correction with a threshold of 0.05. Details of the PR cell types identified across all cohorts are provided in Table S7.

### Computation of PCCs in paired single-cell and bulk RNA-seq analyses

In the analysis of the GSE176078 dataset, we primarily utilized two evaluation metrics to calculate the discrepancy between the deconvolution results and the ground truth: the sample-level PCC and the cell-type-level PCC. The sample-level PCC quantifies the correlation between the estimated and true cell-type proportions within a given sample. Let the estimated proportions be

1 $$\widehat{p}=[{\widehat{p}}_{1},{\widehat{p}}_{2},\dots ,{\widehat{p}}_{k}]$$

and the true proportions be

2 $$p=[{p}_{1},{p}_{2},\dots ,{p}_{k}]$$

where $k$ is the number of cell types. The sample-level PCC is computed as:

3 $${PCC}_{sample}=\frac{{\sum }_{i=1}^{k}({\widehat{p}}_{i}-\overline{\widehat{p}})({p}_{\mathrm{i}}-\overline{p})}{\sqrt{{{\sum }_{i=1}^{k}({\widehat{p}}_{i}-\overline{\widehat{p}})}^{2}}\sqrt{{{\sum }_{i=1}^{k}({p}_{i}-\overline{p})}^{2}}}$$

where $\overline{\widehat{p} }$ and $\overline{p }$ are the means of the estimated and true proportions across all cell types in that sample.

The cell-type-level PCC evaluates, for a given cell type, the correlation between estimated and true proportions across all samples.

Let the estimated proportions across $n$ samples be

4 $$\widehat{p}=[{\widehat{p}}_{1},{\widehat{p}}_{2},\dots ,{\widehat{p}}_{n}]$$

and the true proportions be

5 $$p=\left[{p}_{1},{p}_{2},\dots ,{p}_{n}\right]$$

where $n$ is the number of samples. The cell-type–wise PCC is defined as:

6 $${PCC}_{celltype}=\frac{{\sum }_{i=1}^{n}({\widehat{p}}_{i}-\overline{\widehat{p}})({p}_{i}-\overline{p})}{\sqrt{{{\sum }_{i=1}^{n}({\widehat{p}}_{i}-\overline{\widehat{p}})}^{2}}\sqrt{{{\sum }_{i=1}^{n}({p}_{i}-\overline{p})}^{2}}}$$

where $\overline{\widehat{p} }$ and $\overline{p }$ denote the means of the estimated and true proportions across all samples.

### Evaluation metrics in benchmark

#### Scenario 1: consistency of DP cell types with scRNA-seq

Conservative and permissive F1-scores were used to evaluate the consistency between deconvolution results and scRNA-seq-defined differentially proportioned (DP) cell types, calculated using the formula:

7 $${F1}_{ }=2\times \frac{Precision\times Recall}{Precision+Recall}.$$

Recall represents the fraction of scRNA-seq-defined DP cell types that were also identified by deconvolution, which is consistent for both conservative and permissive criteria. Precision, however, is defined differently under the two criteria. Under conservative criteria, precision corresponds to the fraction of deconvolution-based DP cell types that are validated by scRNA-seq (i.e., DP cell types in scRNA-seq). Under permissive criteria, precision includes cell types with consistent trends in the same direction of proportional change in scRNA-seq, even if they do not reach statistical significance.

#### Scenario 2: reproducibility of DP cell types across cohorts

Conservative and permissive Jaccard idex measured the reproducility of DP cell types across cohorts, i.e., the fraction of shared DP cell types among the union of DP cell types of paired bulk cohorts. Shared DP cell types are clearly defined under conservative criteria, while under permissive criteria, a DP cell type is considered shared if it exhibits a statistically significant proportional change in one cohort, with the same direction of proportional change observed in the other cohort (regardless of statistical significance in the latter). Additionally, F1-scores were used to assess the consistency of shared DP cell types against scRNA-seq-defined DP cell types. In Scenario 2, both the conservative F1-score and permissive F1-score were calculated based on the permissive F1-score approach from Scenario 1. The difference between them lies in the variation of shared DP cell types under the conservative and permissive criteria.

#### Scenario 3: reproducibiltity of PR cell types acrss cohorts

The conservative and permissive Jaccard index was used to evaluate the consistency of PR cell types across bulk datasets. Shared PR cell types between cohorts were defined in the same way as shared DP cell types in Scenario 2.

### Pan-cancer single-cell atlas construction and visualization

We aggregated single cells from the scRNA-seq datasets of 10 cancer types, generating a comprehensive pan-cancer atlas comprising 1.05 million cells. Based on the original cell type annotations provided by the authors, cells were grouped into eight major types: epithelial cells, cancer cells, tissue-specific cells, T & NK cells, myeloid cells, fibroblasts, endothelial cells, and B cells.

To harmonize subtype annotations across datasets for the five major immune and stromal cell types (T & NK cells, myeloid cells, fibroblasts, endothelial cells, and B cells), we combined all cells of each type, normalized expression using Seurat [62] v4.4.0’s NormalizeData(), selected 2,000 variable genes with FindVariableFeatures(), and corrected batch effects via IntegrateData(). Principal component analysis (PCA) was performed using RunPCA() (top 30 components), followed by unsupervised clustering (FindNeighbors(), FindClusters()) and UMAP visualization (RunUMAP()). Clusters were annotated based on published marker genes [30, 63–66] and considered distinct cell subtypes, with the marker gene list provided in Fig. 5B. Across the five major cell types, we identified 33 subtypes: 9 T & NK, 8 myeloid, 6 fibroblast, and 5 each for B cells and endothelial cells. For pan-cancer deconvolution analyses, these 33 subtypes, together with the three unsplit cell types (epithelial, cancer, and tissue-specific cells), were collectively used.

### GO enrichment analysis

Marker genes distinguishing mCAF from other fibroblast subtypes were identified with Seurat’s FindMarkers() (|log2FC|> 1, adj.p.val < 0.05). GO enrichment analysis was performed with clusterProfiler [67] v4.10.0’s enrichGO().

### Statistical tests

Wilcoxon tests were performed using stat_compare_means() from ggpubr v0.6.0 R package, Fisher’s tests with fisher.test() from stats v4.3.3 R package, and hierarchical clustering using pheatmap() from pheatmap v1.0.12 R package. Wilcoxon signed-rank test were performed using wilcox.test() from stats v4.3.3 R package. Multivariate Cox regression for survival analysis was conducted with the coxph() function from the survival v3.5 R package. Kaplan–Meier plots were generated using the ggsurvplot() function from survminer v0.4.9 R package.

## Supplementary Information

Additional file 1: Fig. S1. Heatmap of the performance rankings of the five deconvolution methods across all cell types in the GSE176078 dataset. Fig. S2. Reference-dependence bias of Bisque. Fig. S3. Comparison of deconvolution results for B cell proportions among different methods in GEO-COAD versus B cell proportions in COAD scRNA-seq data. Fig. S4. Deconvolution results of five deconvolution methods for NK cell type in the GEO-COAD dataset. Fig. S5. Performance of the five deconvolution methods across metrics used for F1-score calculation in Scenario one. Fig. S6. Impact of minor cell types on performance evaluation. Fig. S7. Performance of the five deconvolution methods across metrics used for F1-score calculation in Scenario two. Fig. S8. Reproducibility of prognostic-related cell type identification for each deconvolution methods when the FDR threshold was set to 0.1 and 0.15. Fig. S9. Kaplan–Meier survival curves in 11 TCGA entities for mCAF proportion. Fig. S10. The pan-cancer prognostic analysis results of BayesPrism across 11 cancer entities. Fig. S11. Kaplan–Meier survival curves in 11 TCGA and five GEO entities for prognostic indicators. Fig. S12. Quantitative evaluation of deconvolution performance using median absolute erroranalysis of DP cell-type consistency. Fig. S13. Performance of deconvolution methods in DP-based evaluation using non-cancer datasets. Fig. S14. Impact of bulk cohort platform on performance evaluation. Method performance was assessed separately in microarray and RNA-seq cohorts.

Additional file 2: Table S1. Rankings of deconvolution results for the five deconvolution methods across all pseudobulk and realbulk samples in the GSE176078 dataset. Table S2. Bulk datasets used in the benchmark and pan-cancer analysis. Table S3. scRNA-seq datasets used in the benchmark and pan-cancer analysis. Table S4. The cell type annotations used for each cancer type in the benchmark. Table S5. Datasets besides benchmark evaluation and pan-cancer analysis. Table S6. All DP cell types detected by deconvolution methods and their corresponding p-values in scRNA-seq data. For deconvolution results, the FDR threshold was set at 0.05; for DP cell types in scRNA-seq data, the FDR threshold was 0.1. Condition 1 and Condition 2 correspond to those in Table S2. Table S7. All PR cell types detected by deconvolution methods.

## Data Availability

The download links or GEO accession numbers for all scRNA-seq and bulk RNA-seq data used in this study are listed in **Table S2** - **5.**. All code (under a MIT license) and related data files for the analyses are publicly available on GitHub at https://github.com/TianLab-Bioinfo/Benchmark-realdata [68] and https://zenodo.org/records/18153909 [69].
