# Supplementary material for: Evaluating deconvolution methods using real bulk RNA-expression data for robust prognostic insights across cancer types
Source: Genome Biol. 2026 Jan 21;27:38. doi: 10.1186/s13059-026-03942-1 (PMC12906006; doi:10.1186/s13059-026-03942-1)
Supplement: Supplementary file 1 — Additional file 1: Fig. S1. Heatmap of the performance rankings of the five deconvolution methods across all cell types in the GSE176078 dataset. Fig. S2. Reference-dependence bias of Bisque. Fig. S3. Comparison of deconvolution results for B cell proportions among different methods in GEO-COAD versus B cell proportions in COAD scRNA-seq data. Fig. S4. Deconvolution results of five deconvolution methods for NK cell type in the GEO-COAD dataset. Fig. S5. Performance of the five deconvolution methods across metrics used for F1-score calculation in Scenario one. Fig. S6. Impact of minor cell types on performance evaluation. Fig. S7. Performance of the five deconvolution methods across metrics used for F1-score calculation in Scenario two. Fig. S8. Reproducibility of prognostic-related cell type identification for each deconvolution methods when the FDR threshold was set to 0.1 and 0.15. Fig. S9. Kaplan–Meier survival curves in 11 TCGA entities for mCAF proportion. Fig. S10. The pan-cancer prognostic analysis results of BayesPrism across 11 cancer entities. Fig. S11. Kaplan–Meier survival curves in 11 TCGA and five GEO entities for prognostic indicators. Fig. S12. Quantitative evaluation of deconvolution performance using median absolute erroranalysis of DP cell-type consistency. Fig. S13. Performance of deconvolution methods in DP-based evaluation using non-cancer datasets. Fig. S14. Impact of bulk cohort platform on performance evaluation. Method performance was assessed separately in microarray and RNA-seq cohorts. [file 13059_2026_3942_MOESM1_ESM.docx]

**Figure Legends**

Fig. S1: Heatmap of the performance rankings of the five deconvolution methods across all cell types in the GSE176078 dataset. Cell types were ordered based on the adjusted p-values obtained from the Wilcoxon tests comparing the two conditions in scRNA-seq data.

Fig. S2: Reference-dependence bias of Bisque. ER+ samples from the paired GSE176078 breast cancer dataset were used. For each sample, the proportion of one abundant cell type (myofibroblast-like CAFs, >10%) and one rare cell type (plasmablasts, <1%) in the scRNA-seq reference was artificially scaled to 2×, 3×, 5×, and 10× of the original by replicating cells of that type. Bulk sample proportions of the corresponding cell types were then estimated using different deconvolution methods. Linear regression assessed correlations between estimated bulk proportions and the manipulated scRNA-seq proportions. (A) Myofibroblast-like CAFs. (B) Plasmablasts.

Fig. S3: Comparison of deconvolution results for B cell proportions among different methods in GEO-COAD versus B cell proportions in COAD scRNA-seq data.

Fig. S4: Deconvolution results of five deconvolution methods for NK cell type in the GEO-COAD dataset. In GEO-COAD, MuSiC and CIBERSORT estimated the NK cell proportion as 0 in 99.0% and 81.9% of samples, respectively.

Fig. S5: Performance of the five deconvolution methods across metrics used for F1-score calculation in Scenario one. (A) Recall. (B) Conservative precision. (C) Permissive precision.

Fig. S6: Impact of minor cell types on performance evaluation. Minor cell types were defined from scRNA-seq data as those with a median proportion <1% in either contrast condition. Scenario one analyses were repeated after excluding minor cell types, and results were compared with the full dataset. (A) Distribution of DP cell types across the eight scRNA-seq datasets used in scenario one (no DP cell types identified in the PRAD dataset). (B-C) F1-scores of different methods with minor DP cell types excluded versus the full set of DP cell types, under conservative (B) and permissive (C) criteria. (D-E) Paired comparison of F1-scores between full and reduced sets of DP cell types across methods, with no significant differences observed (Wilcoxon signed-rank test).

Fig. S7: Performance of the five deconvolution methods across metrics used for F1-score calculation in Scenario two. (A) Conservative recall. (B) Conservative precision. (C) Permissive recall. (D) Permissive precision.

Fig. S8: Reproducibility of prognostic-related cell type identification for each deconvolution methods when the FDR threshold was set to 0.1 and 0.15.

Fig. S9: Kaplan-Meier survival curves in 11 TCGA entities for mCAF proportion, p values were derived from multivariate Cox regression.

Fig. S10: The pan-cancer prognostic analysis results of BayesPrism across 11 cancer entities, missing values indicate that, for the corresponding cell type, the number of deconvolution results with a cell proportion greater than 0.1% was insufficient to support survival analysis (with fewer than 15 death events). Results with -ln(HR) values greater than 1 or less than -1 are displayed as 1 and -1, respectively.

Fig. S11: Kaplan-Meier survival curves in 11 TCGA and five GEO entities for prognostic indicators, p values were derived from multivariate Cox regression. (A) TCGA cancer entities. (B) GEO entities.

Fig. S12: Quantitative evaluation of deconvolution performance using median absolute error (MAE) analysis of DP cell-type consistency. For both consistency (bulk vs. scRNA-seq) and reproducibility (between bulk cohorts) tests, the union of DP cell types from the two cohorts under comparison was used. Log2FC values of DP cell types were then compared by MAE in (A) bulk cohorts versus scRNA-seq and (B) between two bulk cohorts. The performance of deconvolution methods is ranked based on the median of MAE values across all datasets.

Fig. S13: Performance of deconvolution methods in DP-based evaluation using non-cancer datasets (COVID-19 and SLE). (A) Cell-type proportions across disease versus normal conditions from scRNA-seq datasets of SLE and COVID-19, with DP cell types highlighted. (B) Concordance between DP cell types identified from deconvolution and scRNA-seq, quantified by F1-scores under conservative and permissive criteria. (C, D) Reproducibility of DP cell types across independent bulk cohorts of the same disease, assessed by Jaccard index (C) and F1-score (D) under both criteria.

Fig. S14: Impact of bulk cohort platform on performance evaluation. Method performance was assessed separately in microarray and RNA-seq cohorts. (A–B) Comparison of performance across platforms using conservative (A) and permissive (B) F1-scores; no significant differences were observed (Wilcoxon test). (C-F) Performance and ranking of five methods within microarray cohorts by conservative (C) and permissive (E) F1-scores, and within RNA-seq cohorts by conservative (D) and permissive (F) F1-scores.


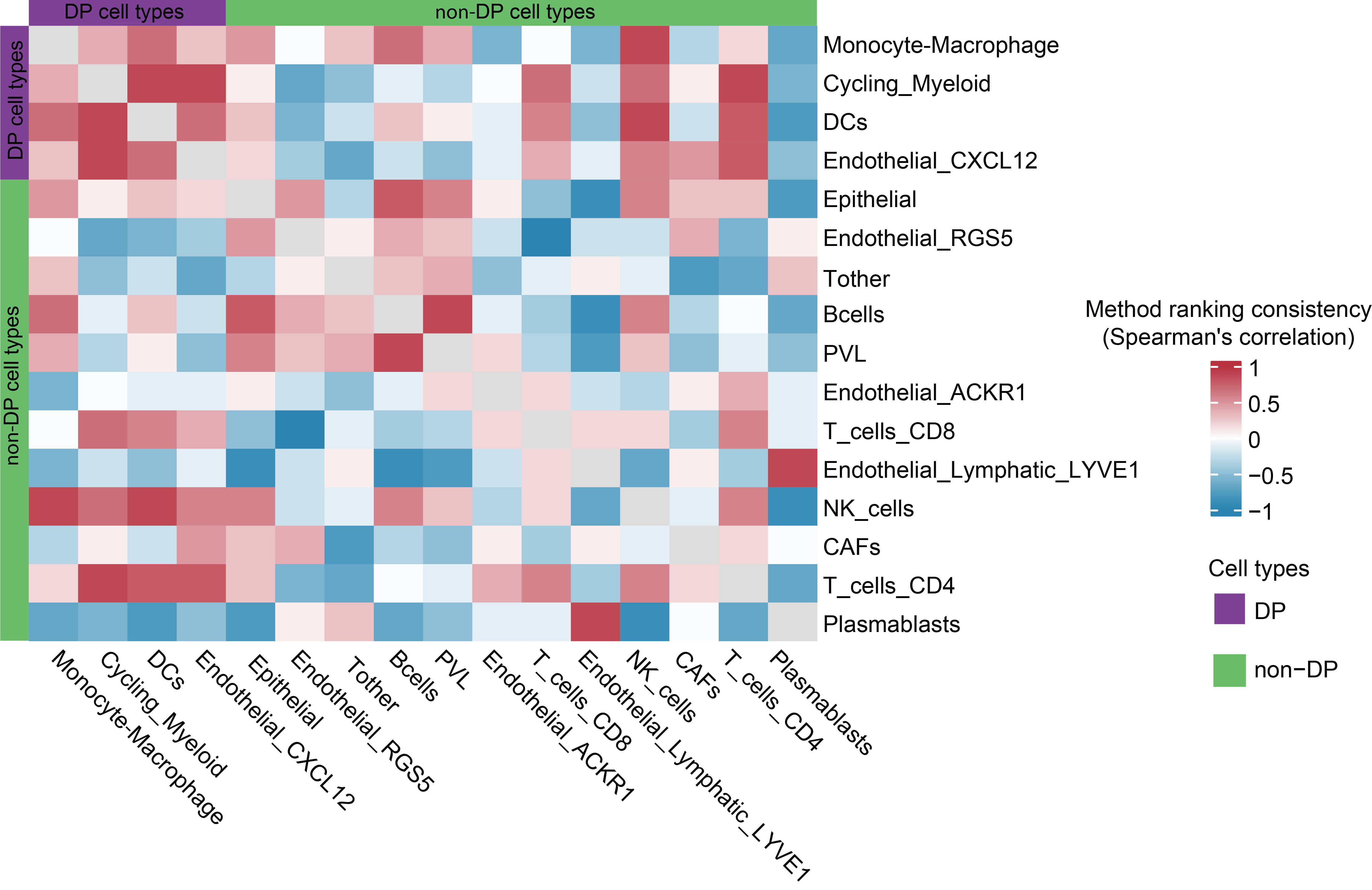


Fig. S1: Heatmap of the performance rankings of the five deconvolution methods across all cell types in the GSE176078 dataset. Cell types were ordered based on the adjusted p-values obtained from the Wilcoxon tests comparing the two conditions in scRNA-seq data.


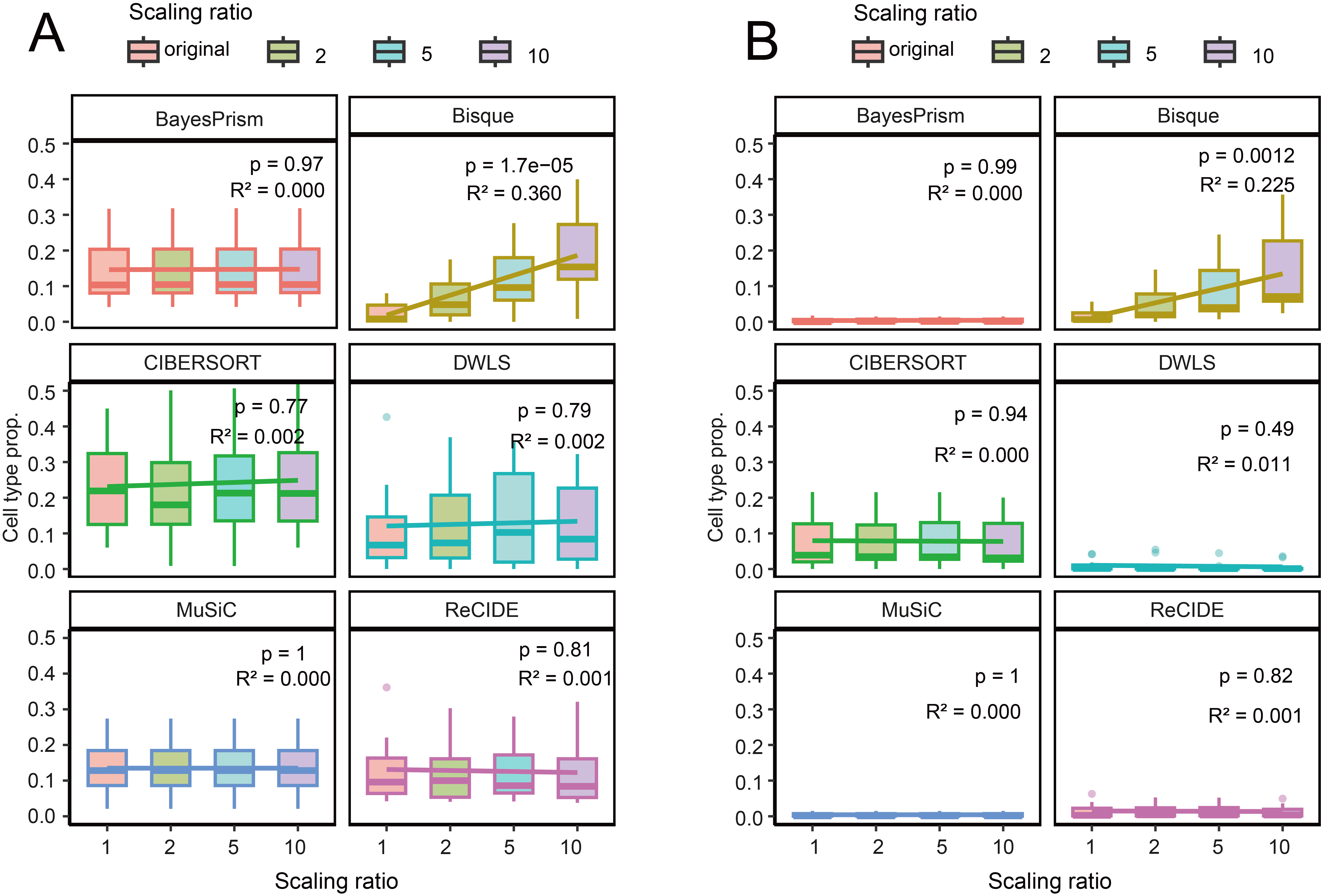


Fig. S2: Reference-dependence bias of Bisque. ER+ samples from the paired GSE176078 breast cancer dataset were used. For each sample, the proportion of one abundant cell type (myofibroblast-like CAFs, >10%) and one rare cell type (plasmablasts, <1%) in the scRNA-seq reference was artificially scaled to 2×, 3×, 5×, and 10× of the original by replicating cells of that type. Bulk sample proportions of the corresponding cell types were then estimated using different deconvolution methods. Linear regression assessed correlations between estimated bulk proportions and the manipulated scRNA-seq proportions. (A) Myofibroblast-like CAFs. (B) Plasmablasts.


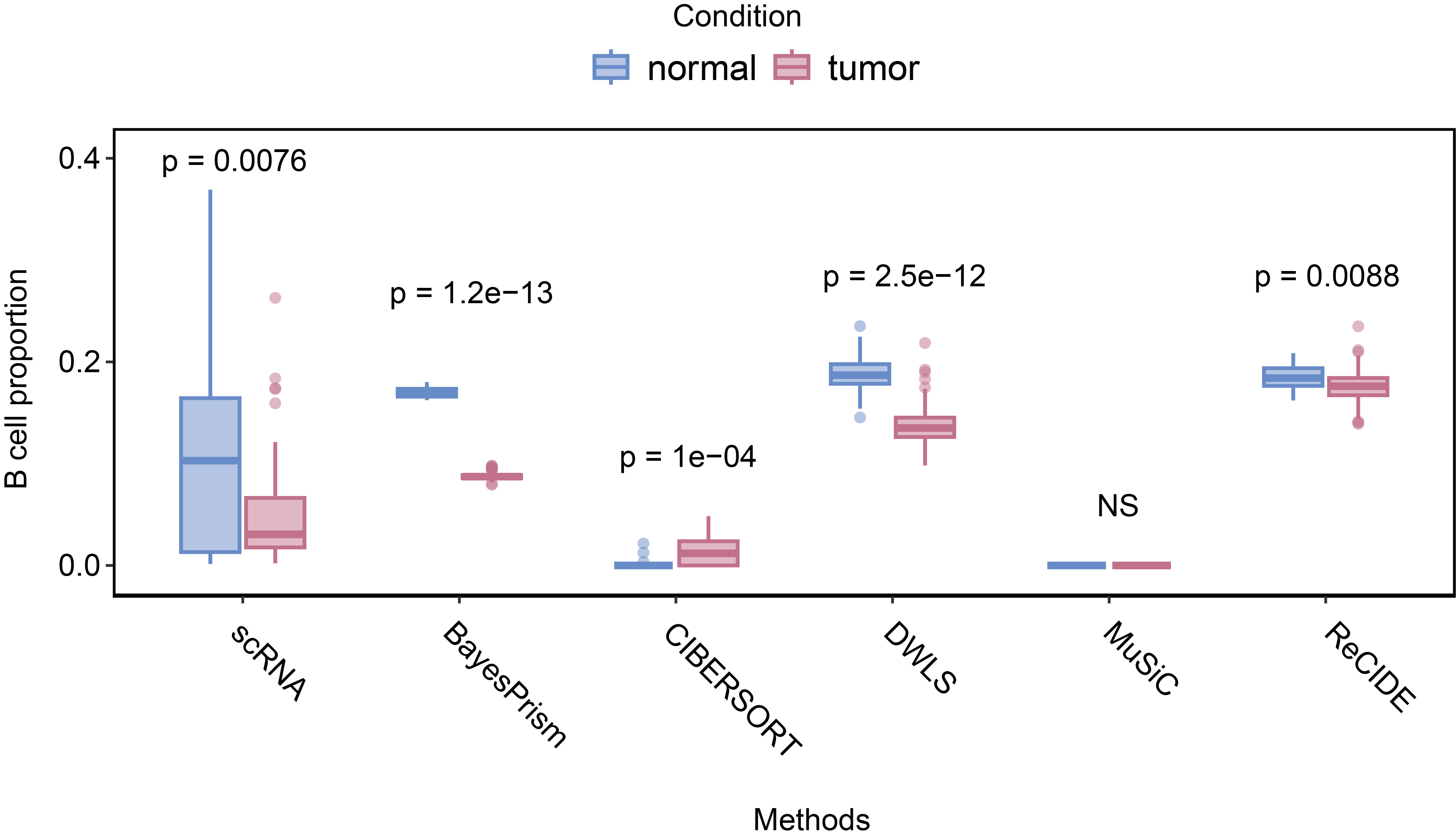


Fig. S3: Comparison of deconvolution results for B cell proportions among different methods in GEO-COAD versus B cell proportions in COAD scRNA-seq data.


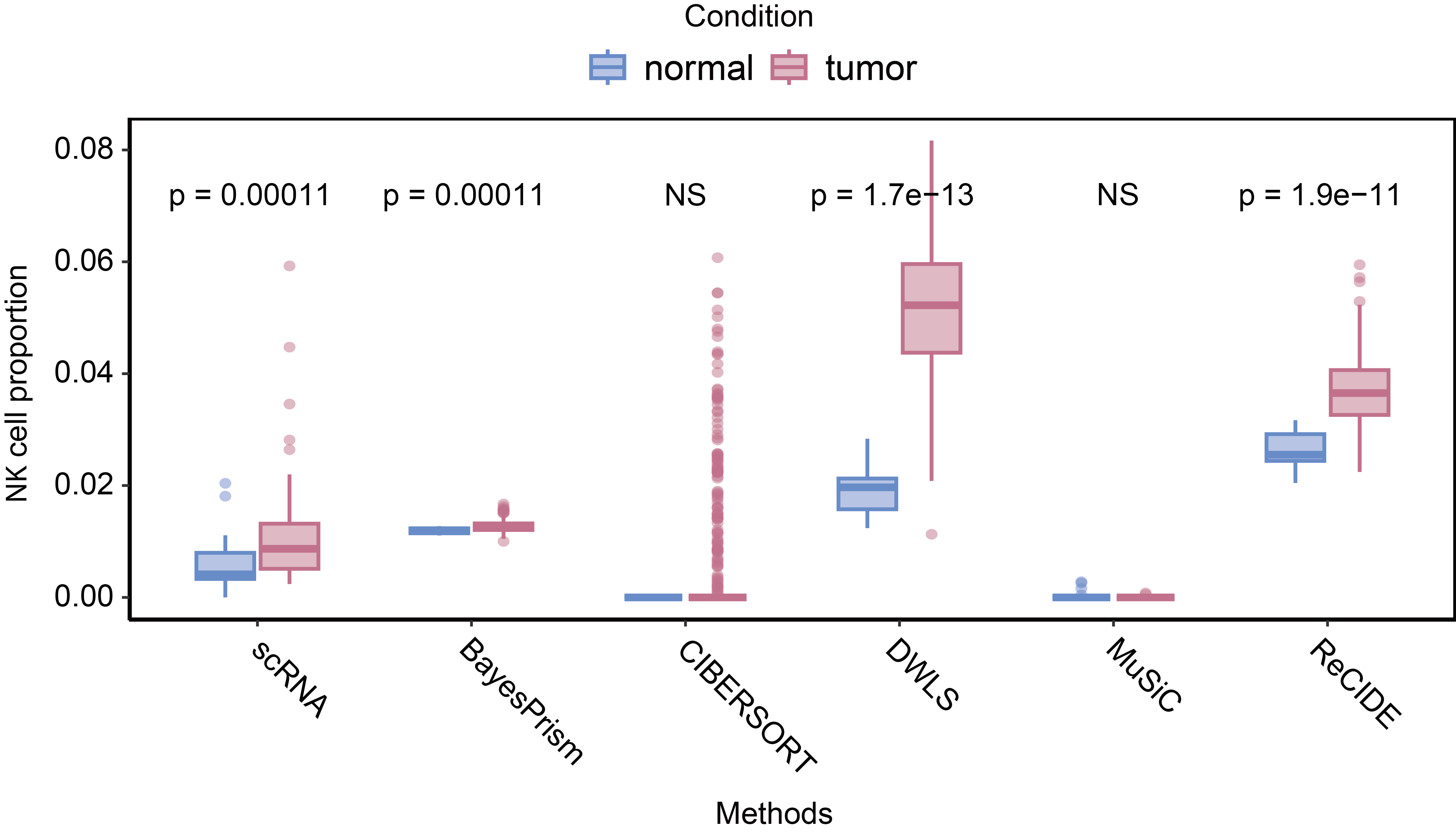


Fig. S4: Deconvolution results of five deconvolution methods for NK cell type in the GEO-COAD dataset. In GEO-COAD, MuSiC and CIBERSORT estimated the NK cell proportion as 0 in 99.0% and 81.9% of samples, respectively.


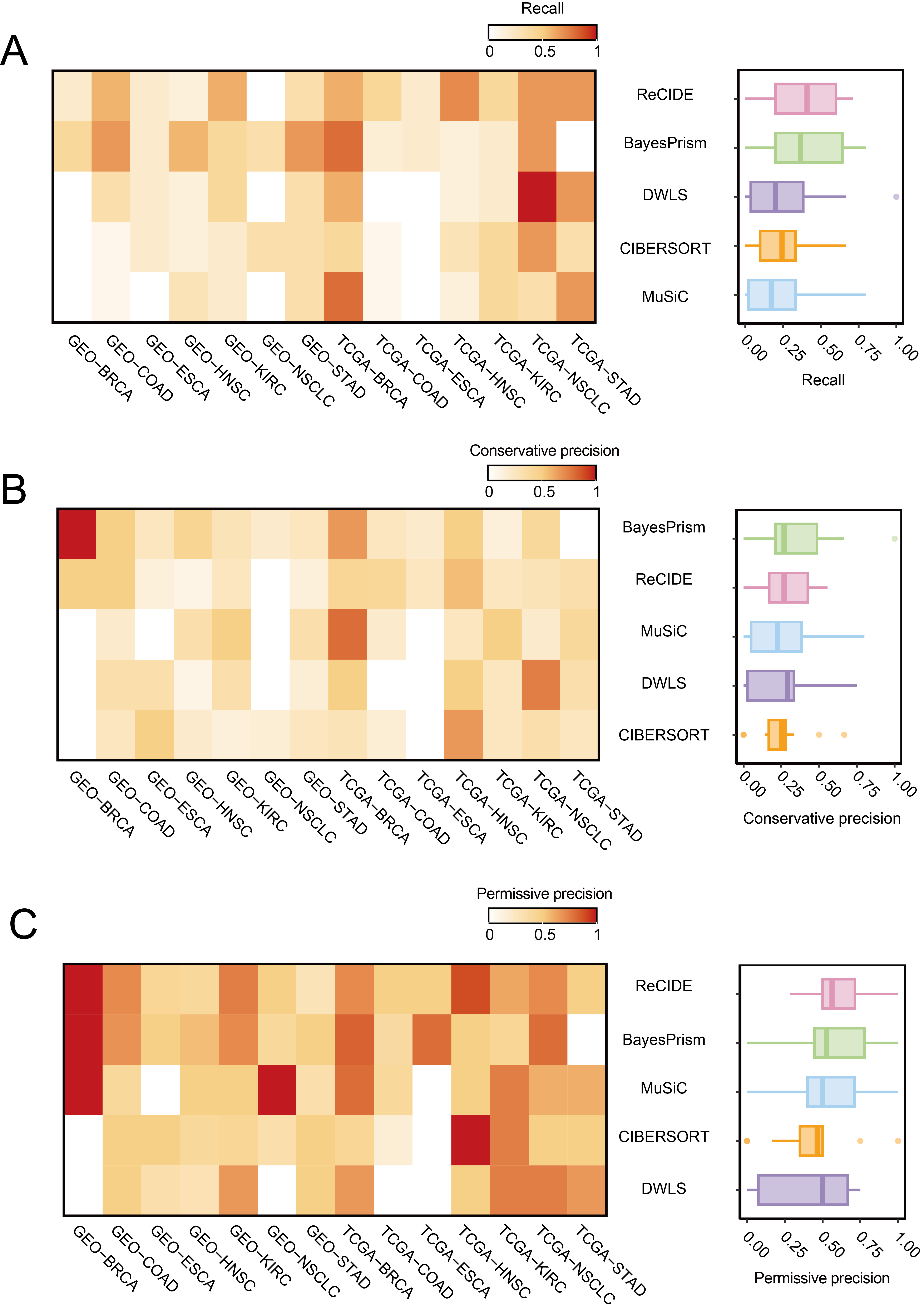


Fig. S5: Performance of the five deconvolution methods across metrics used for F1-score calculation in Scenario one. (A) Recall. (B) Conservative precision. (C) Permissive precision.


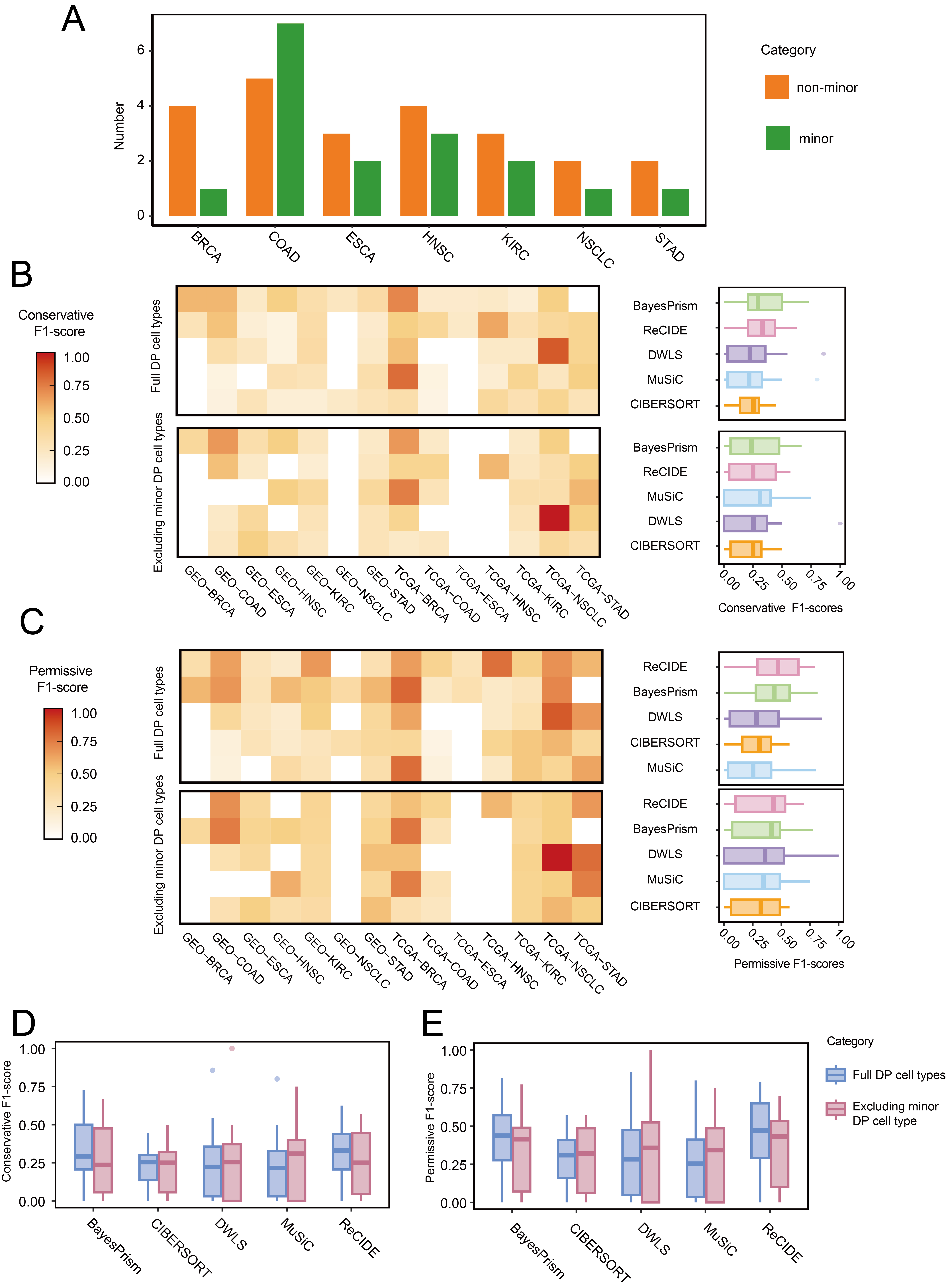


Fig. S6: Impact of minor cell types on performance evaluation. Minor cell types were defined from scRNA-seq data as those with a median proportion <1% in either contrast condition. Scenario one analyses were repeated after excluding minor cell types, and results were compared with the full dataset. (A) Distribution of DP cell types across the eight scRNA-seq datasets used in scenario one (no DP cell types identified in the PRAD dataset). (B-C) F1-scores of different methods with minor DP cell types excluded versus the full set of DP cell types, under conservative (B) and permissive (C) criteria. (D-E) Paired comparison of F1-scores between full and reduced sets of DP cell types across methods, with no significant differences observed (Wilcoxon signed-rank test).


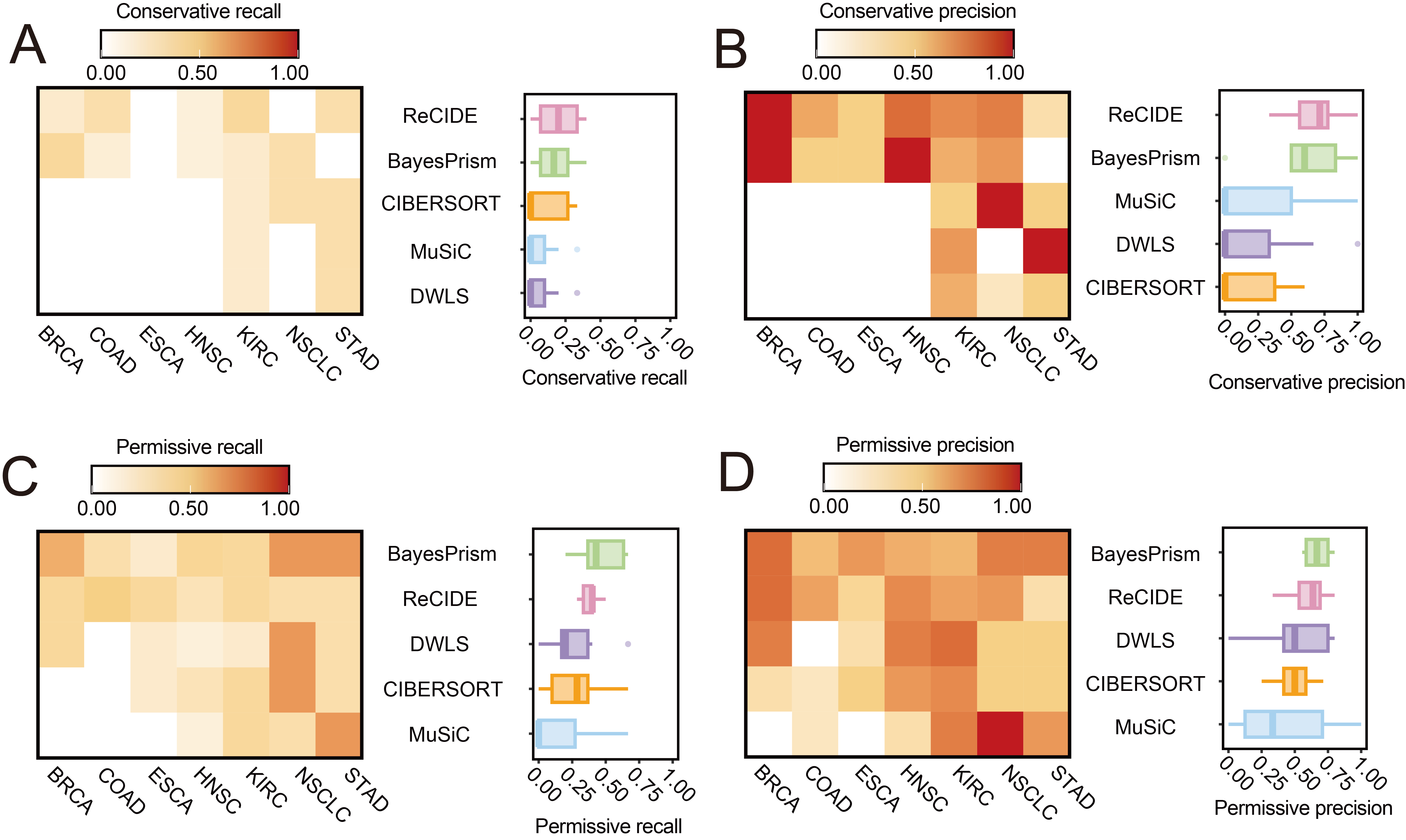


Fig. S7: Performance of the five deconvolution methods across metrics used for F1-score calculation in Scenario two. (A) Conservative recall. (B) Conservative precision. (C) Permissive recall. (D) Permissive precision.


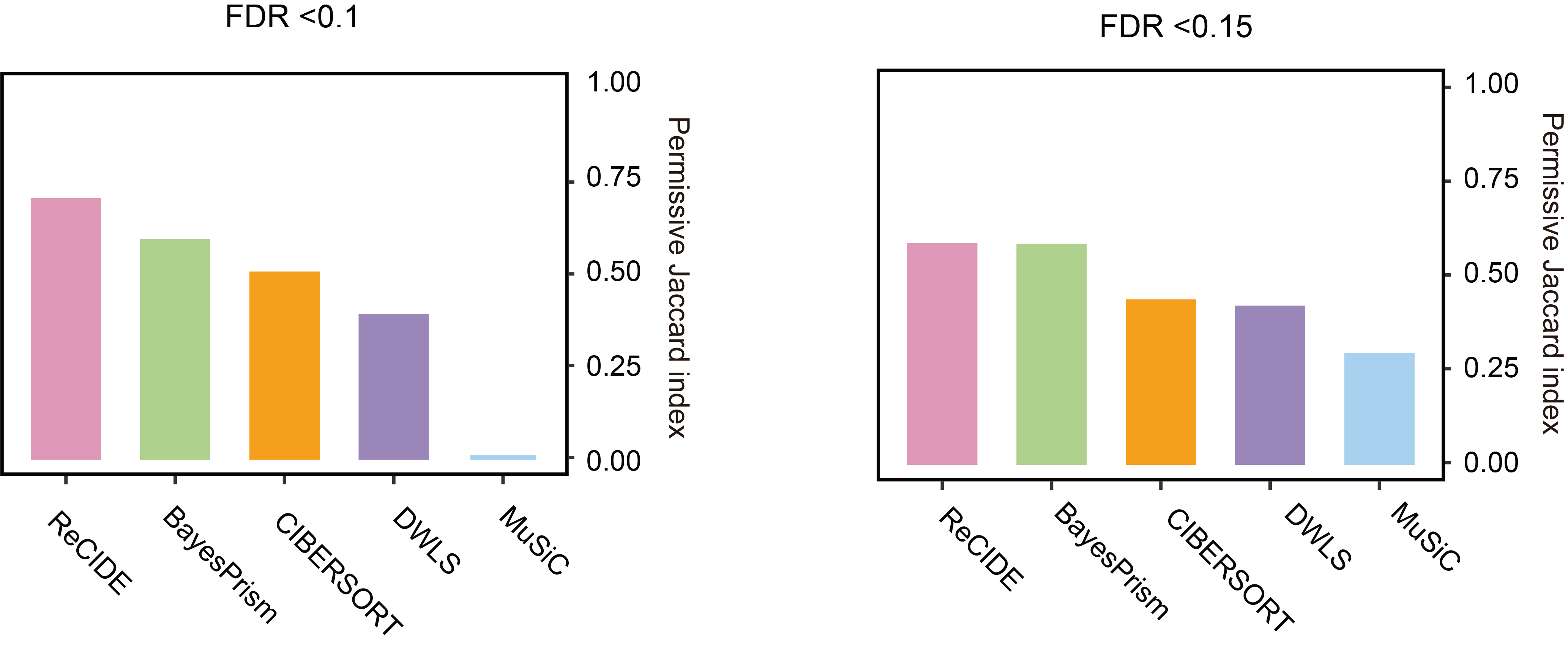


Fig. S8: Reproducibility of prognostic-related cell type identification for each deconvolution methods when the FDR threshold was set to 0.1 and 0.15.


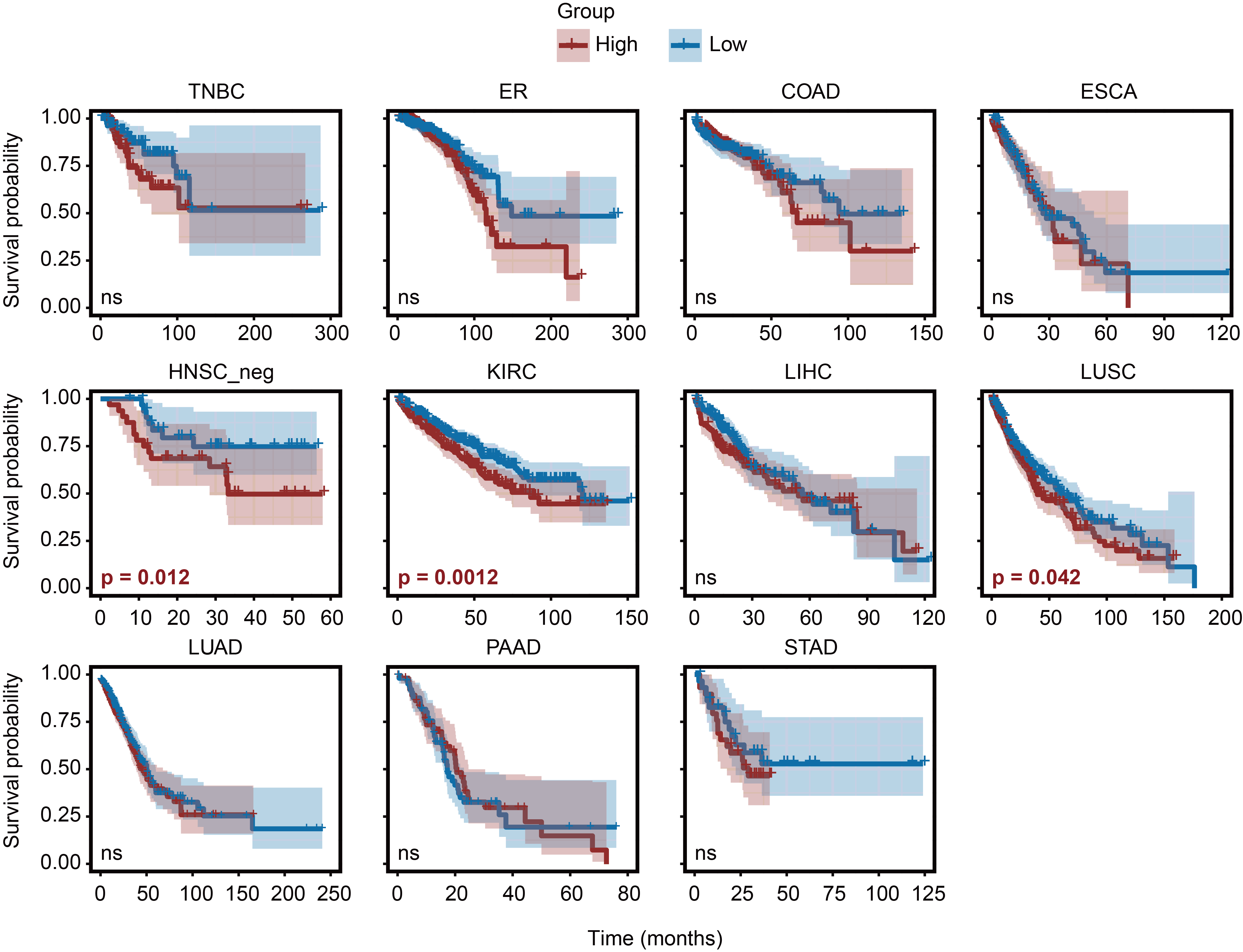


Fig. S9: Kaplan-Meier survival curves in 11 TCGA entities for mCAF proportion, p values were derived from multivariate Cox regression.


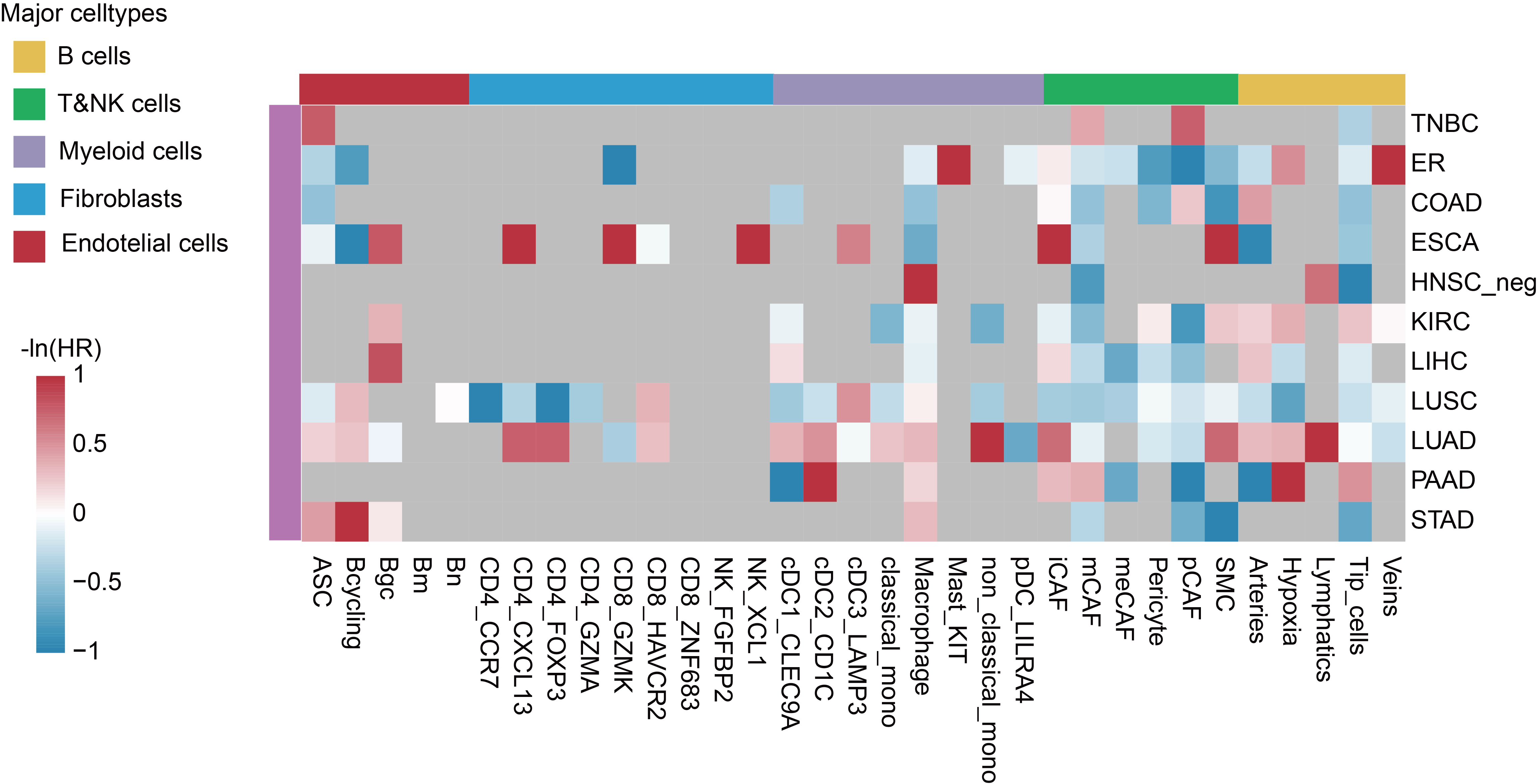


Fig. S10: The pan-cancer prognostic analysis results of BayesPrism across 11 cancer entities, missing values indicate that, for the corresponding cell type, the number of deconvolution results with a cell proportion greater than 0.1% was insufficient to support survival analysis (with fewer than 15 death events). Results with -ln(HR) values greater than 1 or less than -1 are displayed as 1 and -1, respectively.


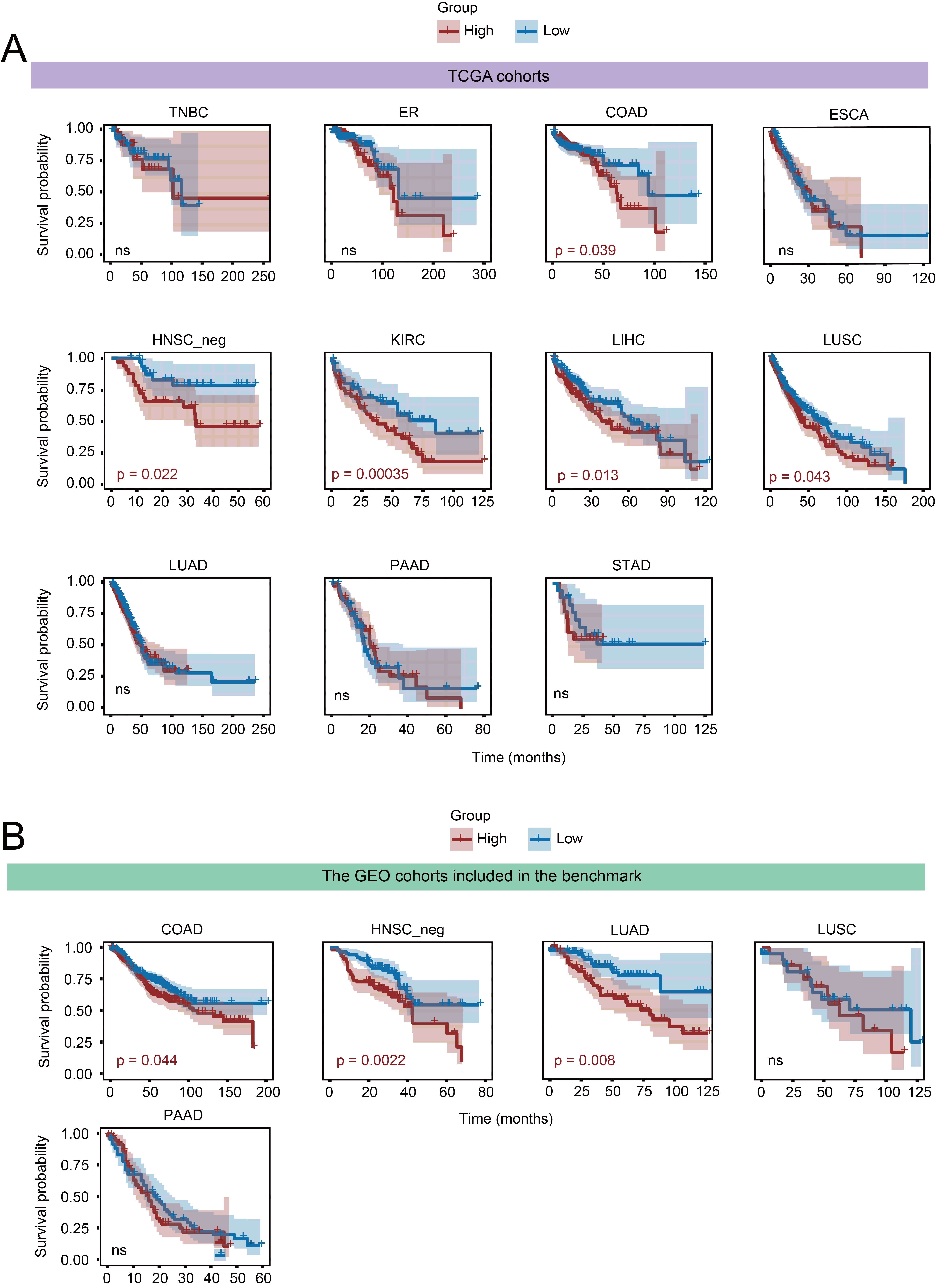


Fig. S11: Kaplan-Meier survival curves in 11 TCGA and five GEO entities for prognostic indicators, p values were derived from multivariate Cox regression. (A) TCGA cancer entities. (B) GEO entities.


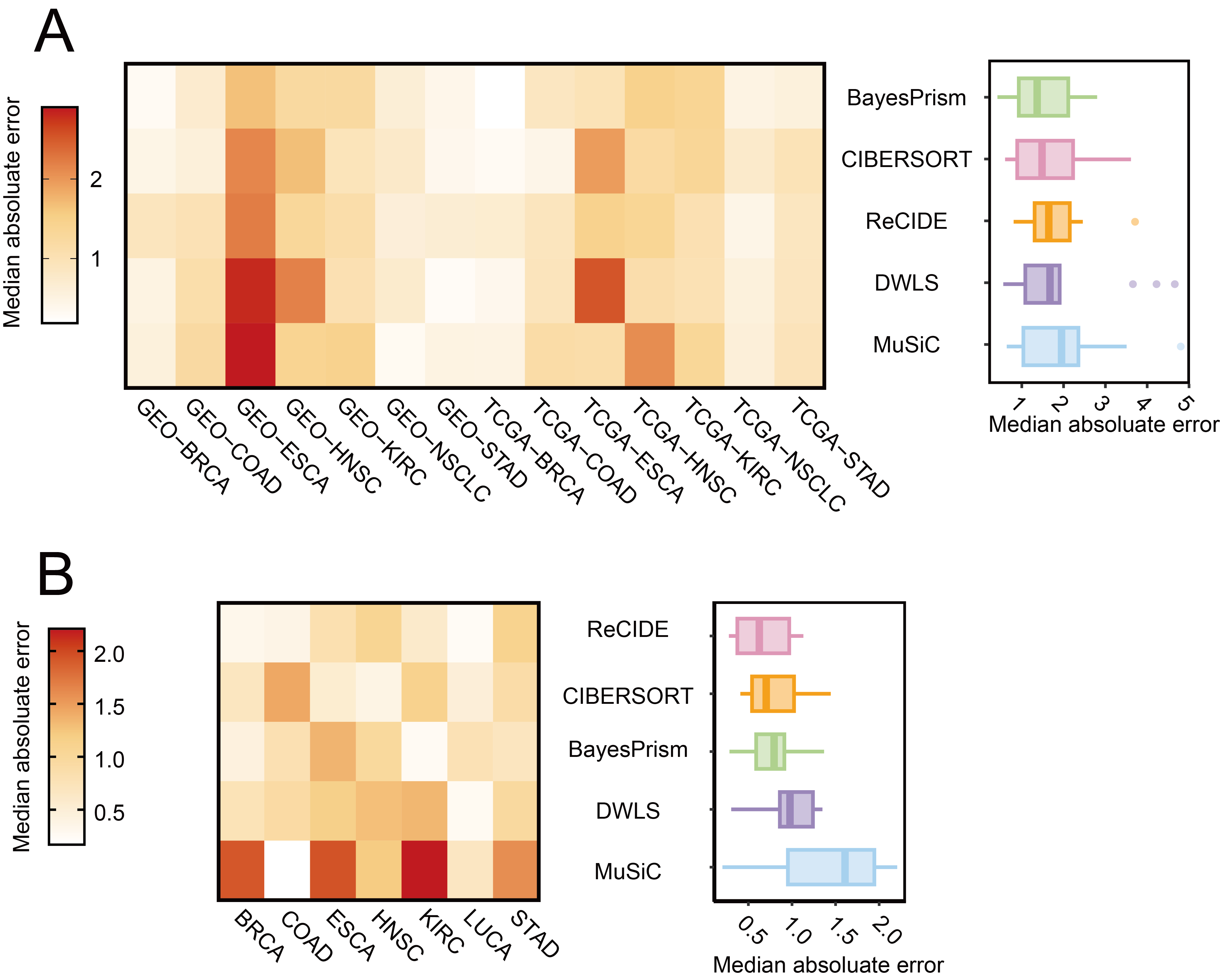


Fig. S12: Quantitative evaluation of deconvolution performance using median absolute error (MAE) analysis of DP cell-type consistency. For both consistency (bulk vs. scRNA-seq) and reproducibility (between bulk cohorts) tests, the union of DP cell types from the two cohorts under comparison was used. Log2FC values of DP cell types were then compared by MAE in (A) bulk cohorts versus scRNA-seq and (B) between two bulk cohorts. The performance of deconvolution methods is ranked based on the median of MAE values across all datasets.
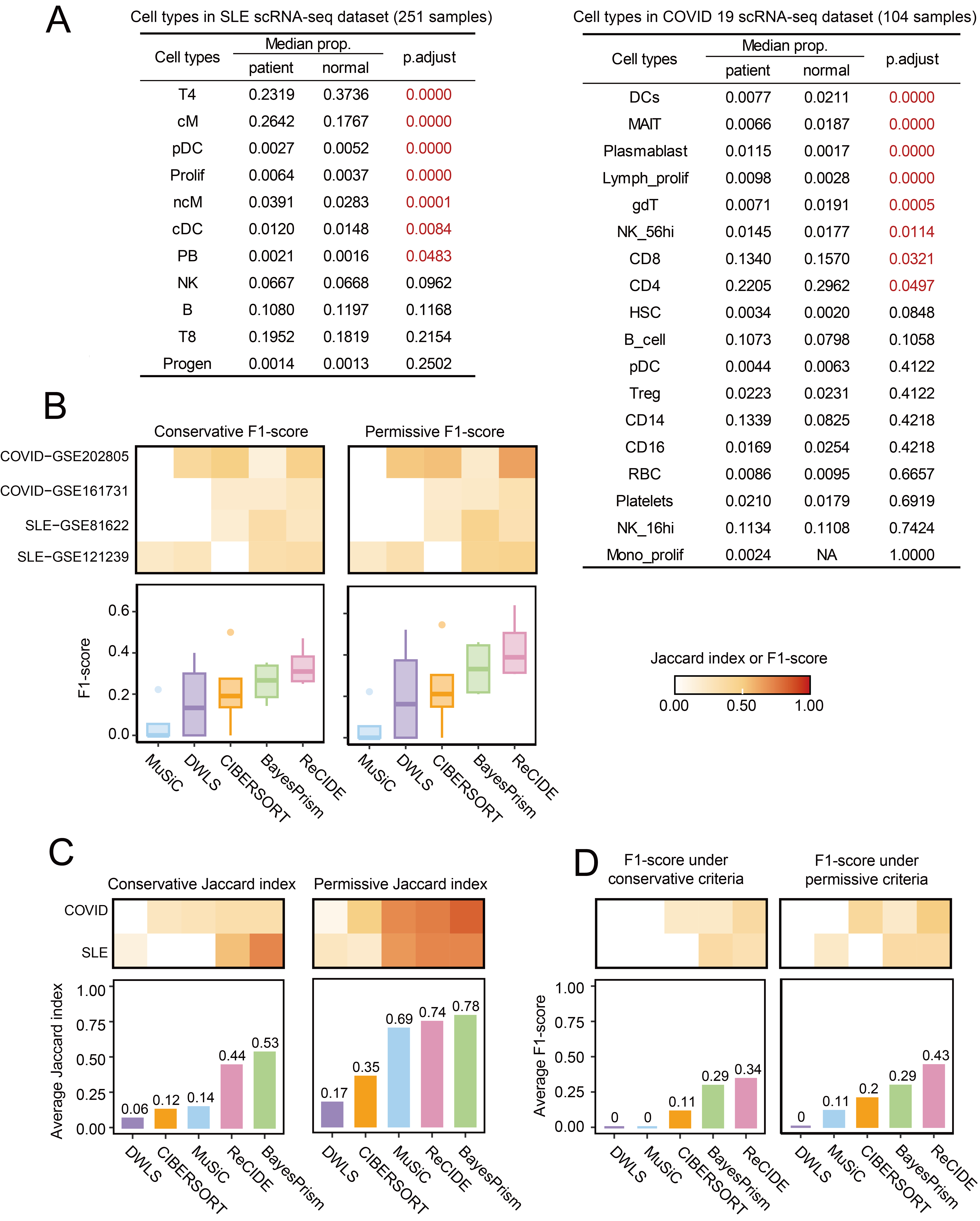


Fig. S13: Performance of deconvolution methods in DP-based evaluation using non-cancer datasets (COVID-19 and SLE). (A) Cell-type proportions across disease versus normal conditions from scRNA-seq datasets of SLE and COVID-19, with DP cell types highlighted. (B) Concordance between DP cell types identified from deconvolution and scRNA-seq, quantified by F1-scores under conservative and permissive criteria. (C, D) Reproducibility of DP cell types across independent bulk cohorts of the same disease, assessed by Jaccard index (C) and F1-score (D) under both criteria.


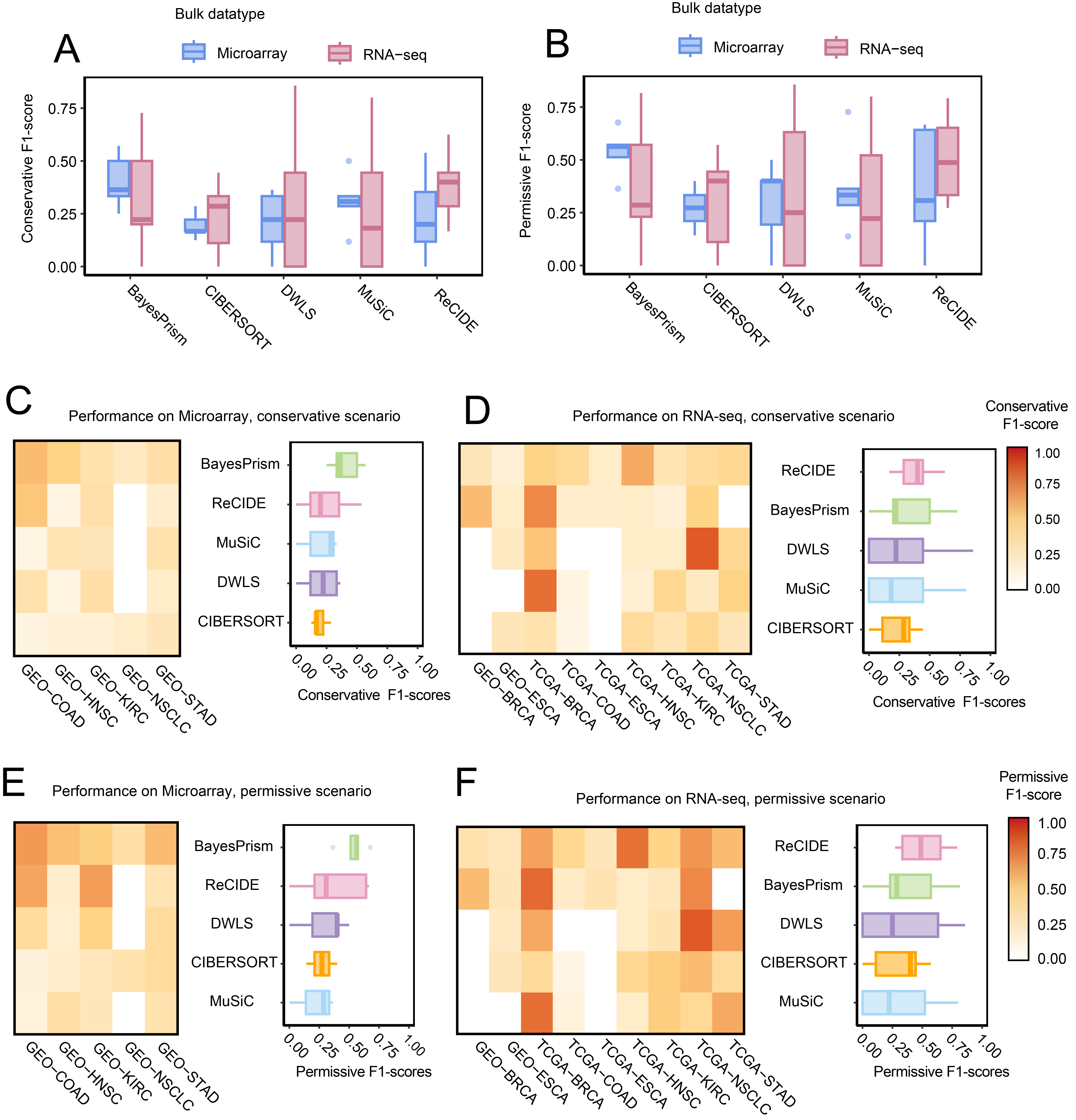


Fig. S14: Impact of bulk cohort platform on performance evaluation. Method performance was assessed separately in microarray and RNA-seq cohorts. (A-B) Comparison of performance across platforms using conservative (A) and permissive (B) F1-scores; no significant differences were observed (Wilcoxon test). (C-F) Performance and ranking of five methods within microarray cohorts by conservative (C) and permissive (E) F1-scores, and within RNA-seq cohorts by conservative (D) and permissive (F) F1-scores.
